# Supplementary material for: Overestimating women’s representation in medicine: a survey of medical professionals’ estimates and their(un)willingness to support gender equality initiatives
Source: BMJ Open. 2022 Mar 3;12(3):e054769. doi: 10.1136/bmjopen-2021-054769 (PMC8943774; doi:10.1136/bmjopen-2021-054769)
Supplement: Author's manuscript [file bmjopen-2021-054769.draft_revisions.pdf]

BMJ Open is committed to open peer review. As part of this commitment we make the peer review history of every article we publish publicly available.

When an article is published we post the peer reviewers' comments and the authors' responses online. We also post the versions of the paper that were used during peer review. These are the versions that the peer review comments apply to.

The versions of the paper that follow are the versions that were submitted during the peer review process. They are not the versions of record or the final published versions. They should not be cited or distributed as the published version of this manuscript.

BMJ Open is an open access journal and the full, final, typeset and author-corrected version of record of the manuscript is available on our site with no access controls, subscription charges or pay-per-view fees (http://bmjopen.bmj.com).

If you have any questions on BMJ Open's open peer review process please email info.bmjopen@bmj.com

BMJ Open

Overestimating women's representation in medicine: A cross-sectional survey of medical professionals' estimates, and their (un)willingness to support gender-equality initiatives

Journal:	*BMJ Open*
Manuscript ID	bmjopen-2021-054769
Article Type:	Original research
Date Submitted by the Author:	22-Jun-2021
Complete List of Authors:	Begeny, Christopher; University of Exeter<br>Grossman, Rebecca; University of Oxford, Endocrinology and Metabolism<br>Ryan, Michelle; University of Exeter; Australian National University, Global Institute for Women's Leadership
Keywords:	HEALTH SERVICES ADMINISTRATION & MANAGEMENT, Human resource management < HEALTH SERVICES ADMINISTRATION & MANAGEMENT, Organisational development < HEALTH SERVICES ADMINISTRATION & MANAGEMENT

SCHOLARONE™
Manuscripts

I, the Submitting Author has the right to grant and does grant on behalf of all authors of the Work (as defined in the below author licence), an exclusive licence and/or a non-exclusive licence for contributions from authors who are: i) UK Crown employees; ii) where BMJ has agreed a CC-BY licence shall apply, and/or iii) in accordance with the terms applicable for US Federal Government officers or employees acting as part of their official duties; on a worldwide, perpetual, irrevocable, royalty-free basis to BMJ Publishing Group Ltd ("BMJ") its licensees and where the relevant Journal is co-owned by BMJ to the co-owners of the Journal, to publish the Work in this journal and any other BMJ products and to exploit all rights, as set out in our licence.

The Submitting Author accepts and understands that any supply made under these terms is made by BMJ to the Submitting Author unless you are acting as an employee on behalf of your employer or a postgraduate student of an affiliated institution which is paying any applicable article publishing charge ("APC") for Open Access articles. Where the Submitting Author wishes to make the Work available on an Open Access basis (and intends to pay the relevant APC), the terms of reuse of such Open Access shall be governed by a Creative Commons licence – details of these licences and which Creative Commons licence will apply to this Work are set out in our licence referred to above.

Other than as permitted in any relevant BMJ Author's Self Archiving Policies, I confirm this Work has not been accepted for publication elsewhere, is not being considered for publication elsewhere and does not duplicate material already published. I confirm all authors consent to publication of this Work and authorise the granting of this licence.

For peer review only

Overestimating women's representation in medicine: A cross-sectional survey of medical professionals' estimates, and their (un)willingness to support gender-equality initiatives

Christopher T. Begeny [1*] Rebecca C. Grossman [2] Michelle K. Ryan [1,3]

*corresponding author

[1] Department of Psychology, University of Exeter, United Kingdom
C.Begeny@exeter.edu

[2] Oxford Centre for Diabetes, Endocrinology and Metabolism, University of Oxford, United Kingdom
rebecca.grossman@bnc.ox.ac.uk

[3] Global Institute for Women's Leadership, Australian National University, Australia
M.Ryan@exeter.ac.uk

Corresponding Author Information
Dr. Christopher T. Begeny
Department of Psychology, University of Exeter
Washington-Singer Laboratories, Perry Road
Exeter, Devon EX4 4QG
United Kingdom

WORD COUNT (incl. abstract, main text, and references): 4,132

Keywords: gender; equality; diversity; medicine; STEMM; stereotypes; bias; women's representation; human resource management, organizational development

For peer review only - http://bmjopen.bmj.com/site/about/guidelines.xhtml

Abstract

Objective

Amidst growing numbers of women in certain areas of medicine (e.g., general practice/primary care), yet their continued underrepresentation in others (e.g., surgical specialties), this study examines: (i) whether medical professionals mistakenly infer that women are now broadly well represented – overestimating women's *true* representation in several different areas and roles; (ii) whether this overestimation of women's representation predicts decreased support for gender-equality initiatives in the field, in conjunction with one's own gender.

Design

Cross-sectional survey

Setting

UK-based medical field

Participants

425 UK medical consultants/general practitioners and trainees (ST/CT1+/SHO/Registrar); 47% female

Main Outcome Measures

Estimates of women's representation in different areas/roles within medicine, examined as a composite estimate and individually; a multi-item measure of support for gender-based initiatives in medicine

Results

Medical professionals tended to overestimate women's true representation in several different areas of medicine (general practice, medical specialties, surgical specialties) and in various roles (consultants/GPs, trainees, medical school graduates). Moreover, these erroneous estimates predicted a decreased willingness to support gender-based initiatives, particularly among men in the field: composite overestimation*respondent-gender interaction, $B = -.04$, 95% CI = -.07 to -.01, $p = .01$. Specifically, while female respondents' (over)estimates were unrelated to their level of support, $B = .00$, 95% CI = -.02 to .02, $p = .92$, male respondents' tendency to overestimate the proportion of women in medicine predicted lower support for gender-based initiatives, $B = -.04$, 95% CI -.06 to -.02, $p < .001$.

For peer review only - http://bmjopen.bmj.com/site/about/guidelines.xhtml

Conclusions

While some progress has been made in gender representation in the medical field, this research illustrates that there are still barriers to gender-equality efforts – and it identifies who within the field is focally maintaining these barriers. It is those individuals (particularly men) who overestimate the *true* progress that has been made in women's representation who are at highest risk for undermining it.

Strengths and Limitations of this Study

1. With women now well represented in some areas of medicine yet underrepresented in others, there remains a dearth of evidence as to whether medical professionals are able to accurately gauge women's representation in different areas/roles; this study helps fill that gap in knowledge.

2. There is also no known evidence as to whether the tendency to overestimate women's true representation can help explain why some medical professionals are reluctant to support gender-equality initiatives in the field.

3. Overall, this research helps medical professionals and related organizations, as well as policymakers, identify barriers to gender-equality efforts – by identifying who within the field may be most likely to resist or withhold support for initiatives that aim to promote gender equality in the field.

4. More broadly, amidst ongoing efforts to promote gender equality in the medical field, this study illustrates that it is important not only to consider the true representation of women in the field but also medical professionals' *perceptions* of women's representation.

5. This study was not poised to discern *why* overestimating women's representation is linked to lower support for gender-equality initiatives (among men; e.g., whether or for whom this overestimation reflects genuine naïveté versus a sense of threat from women's growing numbers in the field).

For peer review only - http://bmjopen.bmj.com/site/about/guidelines.xhtml

Introduction

Paralleling trends in other countries, in the UK women now make up over half of all medical school graduates [1,2]. However, recruitment of female doctors to several specialty areas is not keeping pace with their recruitment to medicine in general [3,4]. For instance, women are well represented in general practice/primary care, yet remain underrepresented in medical and surgical specialties (e.g., in surgical specialties, only 13% of consultants are women) [5].

Despite women's continuing underrepresentation in several areas of medicine, their more prominent representation in general practice and medical schools may be prompting some in the field to mistakenly infer that women are now well represented across the board, or better represented than they actually are in several areas. This is important to consider, partly because if individuals *overestimate* women's representation they may be less willing to support policies and initiatives that aim to further promote gender equality in the profession. They may regard them as no longer necessary, for instance. Indeed, research demonstrates that when individuals overestimate women's representation in a field (e.g., in STEMM), they show less support for initiatives that aim to help women in those fields [6]. Thus, medical professionals who overestimate the true progress that has been made in women's representation in the field may be at highest risk for undermining it.

Medical professionals' tendency to support gender-equality initiatives may hinge on more than their (over)estimates of women in the field, however. It may also depend on medical professionals' own gender. This is partly because gender-based initiatives and related groups (e.g., the General Medical Council Gender Equality Scheme, Women in Surgery at the Royal College of Surgeons) aim to promote not just the representation of women but also the *equal treatment* of women. Thus, representation aside, individuals may continue supporting these initiatives if they are cognizant of ongoing issues with gender bias and discrimination in the field [7–10]. Indeed, recent evidence demonstrates that even when women become well represented in a field, gender biases and unequal treatment persist, and it is predominantly *women* in the field who remain cognizant of this fact (at significantly higher rates than men) [11]. Ultimately, this suggests women in the medical profession may more reliably support gender-based

For peer review only - http://bmjopen.bmj.com/site/about/guidelines.xhtml

initiatives, regardless of their estimations of women's numerical representation in the field, because they are more likely to see the ongoing value in these initiatives for combatting gender bias. By comparison, because men are less likely to recognize issues of gender bias, their support for gender-equality initiatives may more simply, and systematically, vary as a function of their tendency to overestimate women's representation.

Current Research

The current research examines medical professionals' tendency to overestimate women's representation in medicine, and whether such erroneous estimates (along with their own gender) predict a decreased willingness to support gender-based initiatives. Using a sample of UK medical professionals, we first test whether individuals are generally accurate in estimating women's representation in different areas of medicine – general practice, medical and surgical specialties – and in different roles – consultants/GPs, trainees/junior doctors, medical school graduates. We then test whether, as hypothesized, overestimating women's representation predicts decreased support for gender-based initiatives, and whether this is moderated by medical professional's own gender.

Gender-Stereotypical Beliefs about Women in Medicine. As an exploratory step, we also examine individuals' endorsement of a gender-stereotypical belief in men's superiority for the medical profession (e.g., that men are simply better suited for the profession) – a belief that implies women should not be afforded equality in the profession, and thus should predict lower willingness to support gender-equality initiatives [12,13]. Thus, assessing this belief offers two potential insights. First, it allows us to test our core hypothesis – that overestimating women's representation predicts less support for gender-based initiatives, primarily among men – more conservatively, by testing whether this effect (overestimation*respondent-gender interaction) is robust even when accounting for the role of this belief in explaining individuals' (lacking) support for gender-based initiatives. Second, it allows us to assess whether there might be some men, like some women in medicine, who overestimate women's representation yet maintain a consistent level of support for these initiatives. This may be the case among men who more strongly reject this belief (tested via an overestimation*respondent-gender*gender-

For peer review only - http://bmjopen.bmj.com/site/about/guidelines.xhtml

stereotypical belief interaction).

Methods

Participants and Procedure

Participants were 425 UK-based consultants/general practitioners (GPs) and trainees/junior doctors (grades: ST/CT1+/SHO/Registrar) in the medical field (47% female; M_{age}=42.63, SD=11.82; role: 13.9/4.5% consultants/trainees in general practice, 24.6/12.0% consultants/trainees in medicine, 7.9/6.7% consultants/trainees in surgery, 7.4% foundation year 1/2 doctors, 23.0% other (e.g., doctors in industry positions, doctors in psychiatry). Respondents completed a brief survey online described as aiming to "better understand individuals' perceptions of doctors within the UK medical profession." We recruited participants via email, disseminated through list-servs maintained by the 24 medical Royal Colleges and Faculties, 214 NHS Trusts, and 46 medical sub-specialty and social societies. We also recruited respondents via social media and a doctors-only web forum. Participation was voluntary (no remuneration). We excluded four respondents because they indicated that they did not work (nor had worked) in the UK, and three for illogical responses (stating that they believed 98-100% of all consultants and trainees, across all areas, were female; final sample size, n = 418; listwise deletion used as necessary; n = 377-418 for all primary analyses [missing data: 0-25 cases for area/role-specific estimates of women's representation, 41 cases for measure of support for gender-based initiatives]). Sensitivity analyses indicated sample size was generally adequate (based on lowest n, α=.05, 1-β=.80; for detecting $d \geq .14$ in one-sample t tests [see Table 2], for detecting $f^2 \geq .02$ based on ΔR^2 for the addition of the overestimation*respondent-gender interaction term [see Figure 1]). This research did not entail direct involvement of the public or patients.

Measures

Respondents answered questions measuring the following key constructs, and provided demographic information (e.g., gender, age, general area/role in medicine).

Estimates of Females by Area/Role. To assess respondents' estimates of the proportions of women in different areas/roles, we asked, "What percentage of ___ do you think are female?" with the

following inserted: GP (general practitioner) doctors, trainee GP (general practitioner) doctors (ST/CT1+/SHO/Registrar), consultant doctors in medical specialties, trainee doctors in medical specialties (ST/CT1+/SHO/Registrar), consultant doctors in surgical specialties, trainee doctors in surgical specialties (ST/CT1+/SHO/Registrar), medical school graduates. Respondents answered each of these seven questions on a sliding scale from 0-100%. To calculate the degree to which participants under- or overestimated true proportions, we subtracted the actual proportion of females within each area/role (obtained statistics aligned to the time of data collection 2017; [14,15]) from respondents' estimate. Thus, positive values reflected overestimation.

Support for Gender-based Initiatives in the Profession. To assess support for initiatives designed to support women in the UK medical profession, after explaining that such initiatives exist and providing examples (e.g., the General Medical Council Gender Equality Scheme, Women in Surgery at the Royal College of Surgeons) we asked respondents to indicate how much they (dis)agree that these types of initiatives are: necessary, fair, excessive/'over the top' (reverse scored), or put men at a disadvantage (reverse scored). These four items were rated 1-7 (*Strongly Disagree* - *Strongly Agree*), reliable (α = .85), and averaged to form a composite.

Gender-Stereotypical Beliefs about Women in Medicine. To assess endorsement of a gender-stereotypical belief about men's superiority for the medical profession, we asked respondents how much they (dis)agree that, e.g., there is something about being a man that makes one better suited for the medical profession (adapted; [12]). These six items were rated 1-7 (*Strongly Disagree* - *Strongly Agree*), reliable (α = .80), and averaged to form a composite.

For peer review only - http://bmjopen.bmj.com/site/about/guidelines.xhtml

Results

Table 1. Bivariate correlations by gender.

	(above/below diagonal: correlations within female/male respondents, respectively)								
Variable	1	2	3	4	5	6	7	8	9
(Over)estimated % of female:									
1. TRs, general practice	----	.48***	.39***	.49***	.30***	.19**	.61***	.13^{+}	.11
2. TRs, medicine	.51***	----	.39***	.26***	.42***	.26***	.43***	.07	.05
3. TRs, surgery	.20**	27***	----	.32***	.41***	.55***	.45***	.10	-.04
4. DRs, general practice	.64***	48***	.11^{+}	----	.40***	.12^{+}	.33***	.14^{+}	.05
5. DRs, medicine	.21**	.45***	.30***	38***	----	.53***	.35***	.04	-.05
6. DRs, surgery	.16*	27***	.46***	25***	.52***	----	.19**	.05	-.17*
7. Med. school graduates	.61***	48***	.09	53***	.18**	.15*	----	.04	.13^{+}
8. Gender Stereotypical Beliefs	.07	.11	.00	.04	.05	.08	.05	----	-.28***
9. Support for Gender Initiatives	-.15*	-.17*	-.06	-.14^{+}	-.16*	-.18**	-.15*	-.57***	----

TRs = Trainee/junior doctors (ST/CT1+/SHO/Registrar), DRs = GP/Consultant doctors
$^{***}p \leq .001$, $^{**}p \leq .01$, $^{*}p \leq .05$ $^{+}p \leq .10$

Respondent Estimates versus Actual Proportions of Women by Area/Role (Table 2)

We first examined how respondents' estimated proportions of women in different areas/roles compared to actual proportions. Across areas, both male and female respondents tended to overestimate the proportion of female consultants and GPs. Estimated proportions of female trainees varied more by area. As noted in Table 2, these results were also largely evident (among both male and female respondents) when limiting analyses for a given area to the respondents who were themselves in that particular area of medicine. Results also showed that both male and female respondents overestimated the proportion of female medical school graduates.

For peer review only - http://bmjopen.bmj.com/site/about/guidelines.xhtml

Table 2 also shows the standard deviations for each mean estimated proportion. These highlight that, irrespective of the estimated proportion of women in an area/role *on average* (across the sample as a whole), there was substantial variability in estimates *within the sample* of respondents. This variability is key to assessing whether these (over)estimations reliably predict individuals' (lower) levels of support for gender-based initiatives.

Table 2. Respondent estimates versus actual proportions of women by area/role.

Role	Area	Estimated % Female (SD)		Actual % Female	Difference (Est. - Actual)				
Consultants/GPs	General Practice	58.25	(11.49)	54	4.25	[3.15 to 5.36]	$t = 7.57$	$p < .001$ [a]	$d = .37$
	Medicine	43.27	(11.15)	37	6.27	[5.20 to 7.34]	$t = 11.50$	$p < .001$ [a]	$d = .56$
	Surgery	24.99	(10.65)	14	10.99	[9.97 to 12.02]	$t = 21.10$	$p < .001$ [a]	$d = 1.03$
Trainees	General Practice	63.55	(12.35)	69	- 5.45	[-6.68 to -4.23]	$t = -8.75$	$p < .001$	$d = .44$
	Medicine	53.82	(10.15)	53	0.82	[-0.19 to 1.83]	$t = 1.60$	$p = .11$ [a]	$d = .08$
	Surgery	37.37	(11.91)	33	4.37	[3.19 to 5.55]	$t = 7.27$	$p < .001$ [a]	$d = .37$
Medical School Graduates		59.68	(9.83)	55	4.68	[3.70 to 5.65]	$t = 9.44$	$p < .001$	$d = .48$

Positive difference scores indicate overestimations of women's representation; values in brackets are 95% confidence intervals around that difference score; *t*, *p*, and *d* values indicate whether that difference score deviated significantly from zero (one-sample t-test, effect size *d*; i.e., whether estimations of women's representation significantly differed from their true representation); [a] Virtually identical results evident (for both male and female respondents) when limiting analyses to respondents (trainees and consultants/GPs) who were themselves in this area of medicine (analyses not applicable regarding medical school graduates). Actual percentages reflect statistics aligned to the time of data collection (obtained from [14,15]).

Support for Gender-based Initiatives

To test whether respondents' support for gender-based initiatives varied by their tendency to overestimate the proportion of women in medicine and their own gender, we ran moderated regression analyses in SPSS v26 (PROCESS Model 1, 5,000 resamples, gender: 0 *female*, 1 *male*; covariate: age; analyses without covariate evince the same statistically significant results) [16]. Given that the measure of support for gender-based initiatives was not tied to one specific area or role within medicine, it is arguably most relevant to assess how respondents' levels of support varied as a function of their *overall*

For peer review only - http://bmjopen.bmj.com/site/about/guidelines.xhtml

tendency to overestimate women's representation (aggregated across areas/roles). We therefore computed a composite score (*M*=3.84, *SD*=7.47) reflecting respondents' average tendency to overestimate women's representation across the seven aforementioned areas/roles (α=.80 for the seven estimated areas/roles).

As Figure 1 shows, results evinced differences in support for gender-based initiatives as a function of respondents' tendency to overestimate the proportion of women in medicine and their own gender (overestimation*respondent-gender interaction, *B*=-.04, 95% CI=-.07 to -.01, *p*=.01, ΔR^2=.02 for the addition of interaction term, *F*(1,372)=6.48, *p*=.01, f^2=.02; overall *F*(4,372)=8.53, $p < .001$; main effects: overestimation, *B*=-.02, 95% CI=-.04 to -.01, *p*=.01; respondent gender, *B*=-.40, 95% CI=-.64 to -.17, *p*=.001). Tests of simple slopes further showed that female respondents' (over)estimates were unrelated to their level of support (*B*=.00, 95% CI=-.02 to .02, *p*=.92), yet male respondents' tendency to overestimate the proportion of women in medicine predicted lower support for gender-based initiatives (*B*=-.04, 95% CI=-.06 to -.02, $p < .001$).

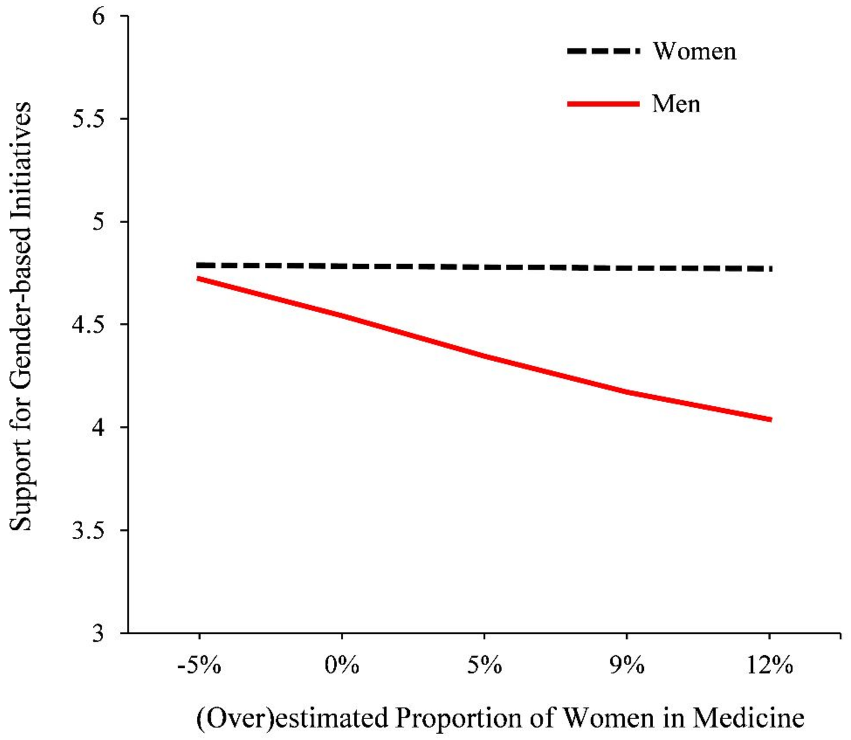

Figure 1. Male and female respondents' (i.e., medical professionals') support for gender-based initiatives in the UK medical profession (1-7 scale), as a function of their estimates of the proportion of women in medicine. Positive values on the x-axis reflect an overestimation of women's representation. Female respondents' estimates were unrelated to their level of support (B = .00, 95% CI = -.02 to .02, p = .92). By comparison, male respondents' tendency to overestimate the proportion of women in medicine predicted significantly less support for gender-based initiatives (B = -.04, 95% CI = -.06 to -.02, p < .001; overestimation*respondent-gender interaction, B = -.04, 95% CI = -.07 to -.01, p = .01, ΔR^2 = .02 for the addition of interaction term, $F(1,372)$ = 6.48, p = .01, f^2 = .02).

We also tested these interaction effects by area/role. As Figure 2 shows, regarding estimates of female trainees in general practice, results showed the same pattern of results (overestimation*respondent-gender interaction, B=-.03, 95% CI=-.04 to -.01, p=.01, ΔR^2=.02 for addition of interaction term, $F(1,372)$=7.13, p=.01; overall $F(4,372)$=7.37, p < .001). Simple slopes showed that female respondents' estimates of female trainees in this area were unrelated to their level of support (B=.01, 95% CI=-.01 to .02, p=.30), yet male respondents' tendency to overestimate the proportion of women in this area predicted less support for gender-based initiatives (B=-.02, 95% CI=-.03 to -.01, p=.01). This same pattern was also found regarding estimates of female trainees in medicine

For peer review only - http://bmjopen.bmj.com/site/about/guidelines.xhtml

(overestimation*respondent-gender interaction, *B*=-.02, 95% CI=-.05 to -.002, *p*=.03; simple slopes: female respondents, *B*=.00, , 95% CI=-.01 to .02, *p*=.71; male respondents *B*=-.02, , 95% CI=-.04 to -.01, *p*=.01), though not for surgery where, notably, women's representation is still quite low (overestimation*respondent-gender interaction, *B*=.00, 95% CI=-.02 to .01, *p*=.65). Regarding estimates of female medical school graduates, results again evinced a significant interaction (overestimation*respondent-gender interaction, *B*=-.03, 95% CI=-.06 to -.01, *p*=.01; simple slopes: female respondents, *B*=.01, 95% CI=-.01 to .02, *p*=.22; male respondents *B*=-.02, 95% CI=-.04 to -.003, *p*=.02).

For peer review only - http://bmjopen.bmj.com/site/about/guidelines.xhtml

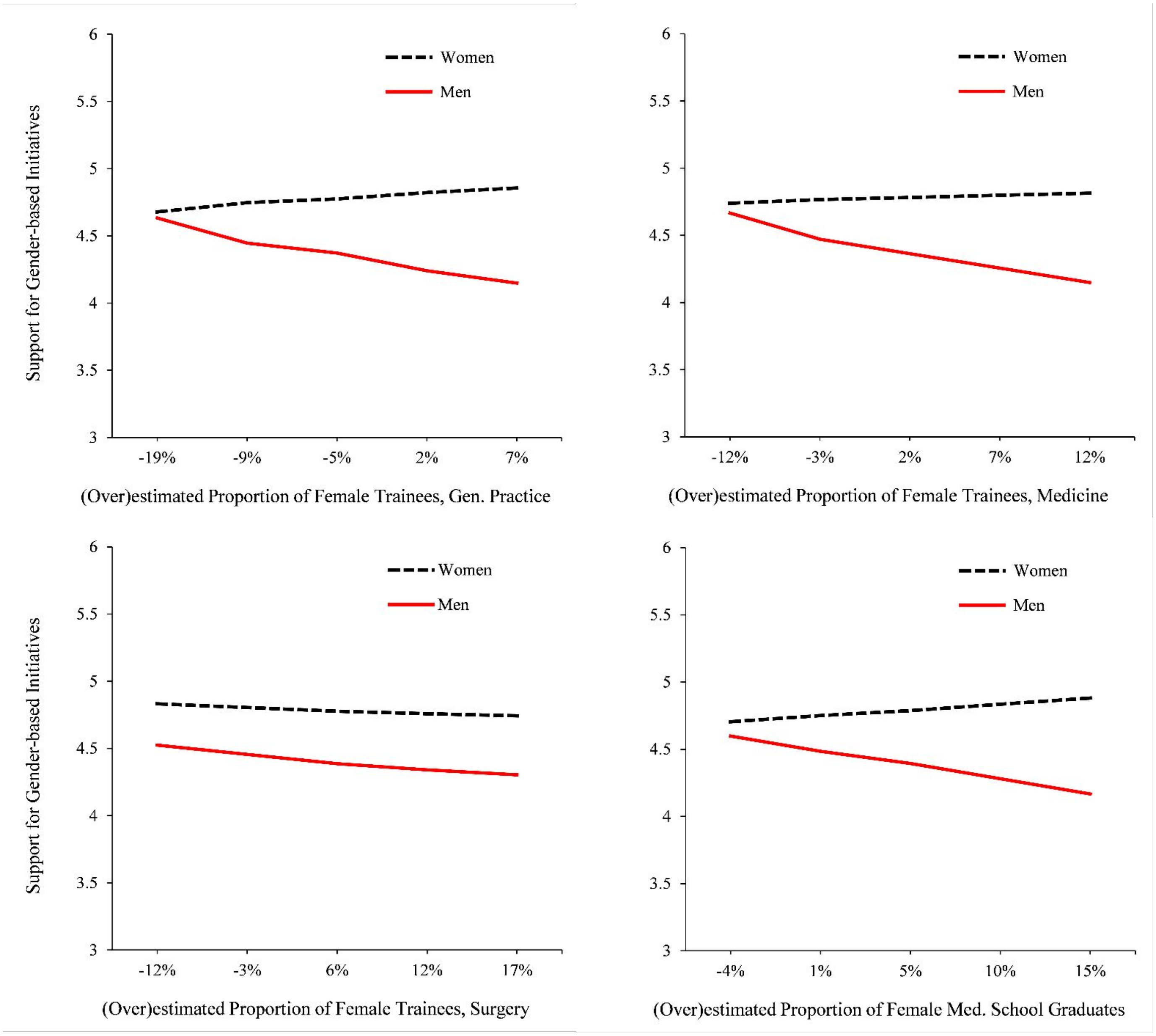

Figure 2. Male and female respondents' (i.e., medical professionals') support for gender-based initiatives in the UK medical profession (1-7 scale), as a function of their estimates of the proportion of: (i) female trainees in general practice, (ii) medicine, and (iii) surgery, and (iv) female medical school graduates. Positive values on the x-axis reflect an overestimation of women's representation in that area/role. In the areas of general practice and medicine, and regarding medical school graduates, female respondents' estimates were unrelated to their level of support, yet male respondents' tendency to overestimate the representation of women in these areas/roles predicted significantly less support for gender-based initiatives. In surgery, neither women's nor men's estimates of female trainees predicted level of support.

For peer review only - http://bmjopen.bmj.com/site/about/guidelines.xhtml

This same pattern of results was also evident when examining respondents' estimates of female GPs/consultants by area, though effects were more modest (overestimation*respondent-gender interactions: general practice, *B*=-.02, 95% CI=-.04 to .00, *p*=.06; medicine, *B*=-.01, 95% CI=-.03 to .01, *p*=.17; surgery, *B*=-.01, , 95% CI=-.03 to .02, *p*=.61). Again, in areas of general practice and medicine (not surgery), female respondents' estimates of female doctors in these areas were unrelated to their level of support (simple slopes for female respondents: general practice, *B*=.00, 95% CI=-.01 to .02, *p*=.81, medicine, *B*=-.01, 95% CI=-.02 to .01, *p*=.35, surgery, *B*=-.02, 95% CI=-.03 to .00, *p*=.05). Yet male respondents' tendency to overestimate the proportion of female doctors in these areas predicted less support for gender-based initiatives (simple slopes for male respondents: general practice, *B*=-.02, 95% CI=-.03 to -.004, *p*=.01, medicine, *B*=-.02, 95% CI=-.04 to -.01, *p*=.01, surgery, *B*=-.02, 95% CI=-.04 to -.01, *p*=.004).

Follow-up Analysis. In follow-up analysis (PROCESS Model 3; paralleling primary analysis using overestimation composite), we tested whether the hypothesized overestimation*respondent-gender effect was robust and/or qualified by respondents' endorsement of the gender-stereotypical belief that men are superior for the medical profession.

Results showed that those who more strongly endorsed this belief had less support for gender-based initiatives (main effects: gender-stereotypical belief: *B*=-.44, 95% CI=-.53 to -.34, $p < .001$; overestimation, *B*=-.01, 95% CI=-.03 to .00, *p*=.06; respondent gender, *B*=-.34, 95% CI=-.55 to -.13, *p*=.001; overall $F(8,362)=18.90$, $p < .001$). Yet at the same time, the hypothesized overestimation*respondent-gender interaction remained significant (*B*=-.04, 95% CI=-.06 to -.01, *p*=.01). Thus, even when accounting for the role of individuals' endorsement of this belief, their level of support for gender-based initiatives still systematically varied by the tendency to overestimate the proportion of women in medicine and their own gender. Results also showed that this interaction was not qualified by a three-way interaction (overestimation*respondent gender*gender-stereotypical belief; *B*=-.01, 95% CI=-.03 to .02, *p*=.70), further illustrating its robustness in explaining individuals' support for gender-based initiatives.

For peer review only - http://bmjopen.bmj.com/site/about/guidelines.xhtml

While the three-way interaction was nonsignificant, the hypothesized effect at different levels of endorsement of this gender-stereotypical belief did illustrate a potentially informative pattern of results. Specifically, male and female respondents who overestimated the proportion of women in medicine but also strongly *rejected* this belief (at the 25th percentile in the belief-endorsement range) did not differ in their level of support for gender-based initiatives (B=-.03, 95% CI=-.07 to .01, p=.14): neither female (B=.01, 95% CI=-.02 to .04, p=.62) nor male (B=-.02, 95% CI=-.05 to .01, p=.11) respondents' tendency to overestimate the proportion of women in medicine predicted less support for initiatives. Yet among those who more strongly *endorsed* this belief (at the 75th percentile), male and female respondents did differ in their support (B=-.04, 95% CI=-.07 to -.01, p =.01): female respondents' overestimates were unrelated to support (B=.00, 95% CI=-.01 to .03, p=.78) while male respondents' overestimates predicted less support for gender-based initiatives (B=-.04, 95% CI=-.06 to -.02, p=.001). Thus, while these analyses were exploratory, they suggest that men who overestimate women's representation may not be invariably more reluctant to support gender-based initiatives. There may be a subset of men who, despite overestimating women's representation, maintain a level of support for gender-based initiatives on par with that of their female counterparts – specifically, those men who more strongly reject the gender-stereotypical belief that men are more suitable for the profession.

Discussion

The strength and quality of the medical profession – including its ability to address an array of public health issues, and to ensure patient satisfaction – hinges on recruiting, retaining and supporting the full range of diverse talent that exists in the population, including among women [10,17]. In this vein, various initiatives are underway to increase women's representation in medicine, with some signs of progress.

Yet amidst this growing gender diversity in medicine – with women now well represented in some areas, yet underrepresented in others – it is important to understand how medical professionals are perceiving this changing demographic landscape. The current research shows that amidst growing numbers of women, medical professionals are tending to overestimate women's true representation, with

For peer review only - http://bmjopen.bmj.com/site/about/guidelines.xhtml

adverse implications. This research shows that when individuals – particularly men – overestimate the proportion of women in medicine they express less support for gender-based initiatives that are striving to promote greater equality. Thus, men who overestimate the *true* progress that has been made in women's representation are at highest risk for undermining it.

This points to an insidious consequence that can arise when women's representation grows within a given field. It seems to prompt some to misperceive and overstate the actual degree of change, and following from this, particularly for men, mistakenly infer that gender-equality initiatives in the field are no longer worth supporting. This ultimately hinders efforts to promote true equality – whether it be promoting women's representation in areas of the field where they are still underrepresented, or combatting issues of gender bias that exist independent of women's numerical representation [11].

In practical terms, this research illustrates the very real nature of the issue – that medical professionals are indeed overestimating women's representation in several areas and roles in the field. Simultaneously, it helps identify *who* within the field is at highest risk for resisting efforts to promote gender equality.

This study does have its limitations. These include uncertainty around the total number of medical professionals who saw the study invitation (given methods for dissemination) and thus the response rate. Additionally, while this study examined estimates of women's representation across seven different key areas and roles, including GPs/consultants and trainees, future research might examine additional roles (e.g., Specialty and Associate Specialist doctors) or specialty areas.

The cross-sectional nature of these data precludes tests of causality. However, previous experimental work supports our hypothesized directionality of effect [12], suggesting that when (male) medical professionals overestimate growth in the number of women in their field it results in less support for gender-based initiatives.

In future research, it will also be important to probe the mechanisms underpinning this overestimation effect. One possibility is that overestimating women's representation prompts individuals, particularly men, to genuinely albeit naïvely infer that gender bias is no longer an issue in their profession

For peer review only - http://bmjopen.bmj.com/site/about/guidelines.xhtml

– that the biases and discrimination that once prevented women from entering the field are no longer occurring (see also [6,11]). As a result, they may regard ongoing gender-based initiatives as unnecessary.

Another possibility is that overestimating women's representation predicts lower support for gender-based initiatives because that overestimation reflects a heightened sense of threat that some men feel, prompting them to exert more resistance to that changing demographic landscape (e.g., expressing less support for gender-based initiatives) [12]. Notably though, our overestimation*respondent-gender effect held true when accounting for individuals' endorsement of the gender-stereotypical belief that men are better suited for the medical profession. This is important because research suggests endorsement of such a belief *reflects* men's sense of threat (i.e., they endorse this type of belief when they feel their high status position in a profession is threatened) [13]. In this way, it seems that an overestimation effect may stand independent of, or is at least not fully explained by, a sense of threat induced by a perceptible growth in women in the field.

Overall, this suggests multiple strategies may be required to address the consequences of this overestimation effect, depending on whether or for whom it is underpinned by a sense of threat versus naïveté about ongoing issues of underrepresentation (if not also ongoing issues of gender bias).

It will also be important to consider whether there are thresholds for spurring this effect. In the current research, we found that while overestimations of women across most areas/roles predicted lower support for gender-equality initiatives, this was not so for surgical specialties (both regarding estimates of GPs/consultants and trainees). This may be because both the actual representation and individuals' overestimations of women in this area are still relatively low (e.g., actual and estimated proportions of female consultants in surgery: 14% and 25%; see Table 2). This suggests that when it is still quite clear that women are vastly underrepresented, aversion to gender-equality initiatives is not piqued – perhaps either because it remains clear that those initiatives are still necessary (from the perspective of a "naïve" over-estimator), or because the still-low representation of women does not yet elicit threat (from the perspective of a "threatened" over-estimator). Going forward, it will also be important to further probe the role of gender in moderating the evinced overestimation effect. One possibility is that this gender-

For peer review only - http://bmjopen.bmj.com/site/about/guidelines.xhtml

moderated effect reflects the fact that men are more likely than women to be unaware of – or simply deny – that gender bias is still an issue in their profession (i.e., in the most precise theoretical terms, it is one's belief that gender bias is no longer an issue, more than gender, that moderates the effect; [11,18]).

Conclusion

Amidst ongoing efforts to promote greater gender equality in medicine, the current research illustrates that it is important not only to consider the true representation of women in the field, but also medical professionals' *perceptions* of women's representation. As shown, individuals' (mis)perceptions are accompanied by growing reservations, or less support for, gender-equality initiatives. In this way, individuals' erroneous estimates mean less support for initiatives that are ultimately working to make the profession *truly* equitable for women.

For peer review only - http://bmjopen.bmj.com/site/about/guidelines.xhtml

References

1 GMC. The state of medical education and practice in the UK: The workforce report. General Medical Council 2019. https://www.gmc-uk.org/about/what-we-do-and-why/data-and-research/the-state-of-medical-education-and-practice-in-the-uk/workforce-report-2019 (accessed 16 Mar 2021).

2 AAMC. The Majority of U.S. Medical Students Are Women, New Data Show. Association of American Medical Colleges 2019. https://www.aamc.org/news-insights/press-releases/majority-us-medical-students-are-women-new-data-show (accessed 16 Mar 2021).

3 RCP. 2016–17 census: UK consultants and higher specialty trainees. Royal College of Physicians 2017. https://www.rcplondon.ac.uk/projects/outputs/2016-17-census-uk-consultants-and-higher-specialty-trainees

4 AAMC. 2020 Physician Specialty Data Report. Association of American Medical Colleges 2020. https://www.aamc.org/data-reports/workforce/interactive-data/active-physicians-sex-and-specialty-2019

5 RCS. Royal College of Surgeons of England, Statistics: Women in Surgery. 2020.https://www.rcseng.ac.uk/careers-in-surgery/women-in-surgery/statistics/

6 Swim JK, Aikin KJ, Hall WS, *et al.* Sexism and racism: Old-fashioned and modern prejudices. *J Pers Soc Psychol* 1995;**68**:199–214. doi:10.1037/0022-3514.68.2.199

7 Burns KEA, Straus SE, Liu K, *et al.* Gender differences in grant and personnel award funding rates at the Canadian Institutes of Health Research based on research content area: A retrospective analysis. *PLOS Med* 2019;**16**:e1002935. doi:10.1371/journal.pmed.1002935

8 Dacre J, Woodhams C. Mend the Gap: The Independent Review into Gender Pay Gaps in Medicine in England. UK Department of Health & Social Care 2020. https://assets.publishing.service.gov.uk/government/uploads/system/uploads/attachment_data/file/944246/Gender_pay_gap_in_medicine_review.pdf

9 Jagsi R, Griffith KA, Stewart A, *et al.* Gender Differences in the Salaries of Physician Researchers. *JAMA* 2012;**307**. doi:10.1001/jama.2012.6183

10 National Academies of Sciences, Engineering, and Medicine. Promising Practices for Addressing the Underrepresentation of Women in Science, Engineering, and Medicine: Opening Doors. Washington, DC: : The National Academies Press 2020. https://www.nap.edu/catalog/25585/promising-practices-for-addressing-the-underrepresentation-of-women-in-science-engineering-and-medicine (accessed 1 Mar 2020).

11 Begeny CT, Ryan MK, Moss-Racusin CA, *et al.* In some professions, women have become well represented, yet gender bias persists—Perpetuated by those who think it is not happening. *Sci Adv* 2020;**6**:eaba7814. doi:10.1126/sciadv.aba7814

12 Danbold F, Huo YJ. Men's defense of their prototypicality undermines the success of women in STEM initiatives. *J Exp Soc Psychol* 2017;**72**:57–66. doi:10.1016/j.jesp.2016.12.014

13 Morton TA, Postmes T, Haslam SA, *et al.* Theorizing gender in the face of social change: Is there anything essential about essentialism? *J Pers Soc Psychol* 2009;**96**:653–64. doi:10.1037/a0012966

14 GMC. The state of medical education and practice in the UK. General Medical Council 2017. https://www.gmc-uk.org/static/documents/content/SoMEP-2017-final-executive-summary.pdf (accessed 16 Mar 2021).

15 NHS Digital. UK National Health Service, HCHS doctors by speciality, grade and gender: April 2015-April 2018. 2018.https://digital.nhs.uk/data-and-information/find-data-and-publications/supplementary-information/2018-supplementary-information-files/staff-numbers/consultants-and-doctors/hchs-doctors-by-speciality.-grade-and-gender-april-2015--april-2018

16 Hayes AF. *Introduction to mediation, moderation, and conditional process analysis: A regression-based approach*. New York, NY, US: : Guilford Press 2013.

For peer review only - http://bmjopen.bmj.com/site/about/guidelines.xhtml

17 Grossman RC. The Gender Pay Gap in Medicine: Causes and Solutions. In: Bellini MI, Papalois VE, eds. *Gender Equity in the Medical Profession*. Hershey, PA: : IGI Global 2020. 110–27.http://doi:10.4018/978-1-5225-9599-1.ch008 (accessed 16 Mar 2021).
18 Moss-Racusin CA, Dovidio JF, Brescoll VL, *et al.* Science faculty's subtle gender biases favor male students. *Proc Natl Acad Sci* 2012;**109**:16474–9. doi:10.1073/pnas.1211286109

For peer review only - http://bmjopen.bmj.com/site/about/guidelines.xhtml

Author Contributions

Conceptualization: CTB, RCG, MKR

Data Curation: CTB, RCG

Formal Analysis: CTB

Funding Acquisition: MKR

Methodology & Design: CTB, RCG, MKR

Project Management & Administration: CTB

Visualization: CTB

Writing, original draft: CTB, RCG

Writing, review & editing: CTB, RCG, MKR

Funding

This work was supported by a European Research Council Consolidator Grant (ERC-CoG 725128) awarded to MKR.

Competing Interests

The authors declare no competing interests. The funders had no role in study design, data collection, analysis or interpretation, nor in the preparation or decision to submit this work for publication.

Ethics Approval

This research was approved by and carried out in compliance with standards for human research set forth by the University of Exeter Ethics Committee (approval for eCLESPsy000134). Informed consent was obtained from participants.

Data Sharing Statement

All data underlying the findings described in this article are available at The Center for Open Science (https://osf.io/hrm63/).

STROBE 2007 (v4) Statement—Checklist of items that should be included in reports of *cross-sectional studies*

Section/Topic	Item #	Recommendation	Reported on page #
**Title and abstract**	1	(*a*) Indicate the study's design with a commonly used term in the title or the abstract	1
		(*b*) Provide in the abstract an informative and balanced summary of what was done and what was found	3-4
**Introduction**			
Background/rationale	2	Explain the scientific background and rationale for the investigation being reported	5-6
Objectives	3	State specific objectives, including any prespecified hypotheses	6
**Methods**			
Study design	4	Present key elements of study design early in the paper	1,3,7
Setting	5	Describe the setting, locations, and relevant dates, including periods of recruitment, exposure, follow-up, and data collection	3,7
Participants	6	(*a*) Give the eligibility criteria, and the sources and methods of selection of participants	7
Variables	7	Clearly define all outcomes, exposures, predictors, potential confounders, and effect modifiers. Give diagnostic criteria, if applicable	3,7-8
Data sources/ measurement	8*	For each variable of interest, give sources of data and details of methods of assessment (measurement). Describe comparability of assessment methods if there is more than one group	7-8
Bias	9	Describe any efforts to address potential sources of bias	6,8,15
Study size	10	Explain how the study size was arrived at	7
Quantitative variables	11	Explain how quantitative variables were handled in the analyses. If applicable, describe which groupings were chosen and why	7-16
Statistical methods	12	(*a*) Describe all statistical methods, including those used to control for confounding	10,15
		(*b*) Describe any methods used to examine subgroups and interactions	10,15
		(*c*) Explain how missing data were addressed	7
		(*d*) If applicable, describe analytical methods taking account of sampling strategy	n/a
		(*e*) Describe any sensitivity analyses	7
**Results**			

For peer review only

Participants	13*	(a) Report numbers of individuals at each stage of study—eg numbers potentially eligible, examined for eligibility, confirmed eligible, included in the study, completing follow-up, and analysed	7
		(b) Give reasons for non-participation at each stage	7
		(c) Consider use of a flow diagram	
Descriptive data	14*	(a) Give characteristics of study participants (eg demographic, clinical, social) and information on exposures and potential confounders	7
		(b) Indicate number of participants with missing data for each variable of interest	7
Outcome data	15*	Report numbers of outcome events or summary measures	7
Main results	16	(*a*) Give unadjusted estimates and, if applicable, confounder-adjusted estimates and their precision (eg, 95% confidence interval). Make clear which confounders were adjusted for and why they were included	10-16
		(*b*) Report category boundaries when continuous variables were categorized	n/a
		(*c*) If relevant, consider translating estimates of relative risk into absolute risk for a meaningful time period	n/a
Other analyses	17	Report other analyses done—eg analyses of subgroups and interactions, and sensitivity analyses	7,15-16
**Discussion**			
Key results	18	Summarise key results with reference to study objectives	16-17
Limitations	19	Discuss limitations of the study, taking into account sources of potential bias or imprecision. Discuss both direction and magnitude of any potential bias	17-18
Interpretation	20	Give a cautious overall interpretation of results considering objectives, limitations, multiplicity of analyses, results from similar studies, and other relevant evidence	16-18
Generalisability	21	Discuss the generalisability (external validity) of the study results	16-18
**Other information**			
Funding	22	Give the source of funding and the role of the funders for the present study and, if applicable, for the original study on which the present article is based	2

*Give information separately for cases and controls in case-control studies and, if applicable, for exposed and unexposed groups in cohort and cross-sectional studies.

Note: An Explanation and Elaboration article discusses each checklist item and gives methodological background and published examples of transparent reporting. The STROBE checklist is best used in conjunction with this article (freely available on the Web sites of PLoS Medicine at http://www.plosmedicine.org/, Annals of Internal Medicine at http://www.annals.org/, and Epidemiology at http://www.epidem.com/). Information on the STROBE Initiative is available at www.strobe-statement.org.

For peer review only

BMJ Open

Overestimating women's representation in medicine: A survey of medical professionals' estimates, and their (un)willingness to support gender-equality initiatives

Journal:	*BMJ Open*
Manuscript ID	bmjopen-2021-054769.R1
Article Type:	Original research
Date Submitted by the Author:	07-Jan-2022
Complete List of Authors:	Begeny, Christopher; University of Exeter, Psychology<br>Grossman, Rebecca; University of Oxford, Endocrinology and Metabolism<br>Ryan, Michelle; Australian National University, Global Institute for Women's Leadership; University of Exeter, Psychology
**Primary Subject Heading**:	Medical management
Secondary Subject Heading:	Communication, General practice / Family practice, Health services research, Medical education and training, Surgery
Keywords:	HEALTH SERVICES ADMINISTRATION & MANAGEMENT, Human resource management < HEALTH SERVICES ADMINISTRATION & MANAGEMENT, Organisational development < HEALTH SERVICES ADMINISTRATION & MANAGEMENT

SCHOLARONE™
Manuscripts

I, the Submitting Author has the right to grant and does grant on behalf of all authors of the Work (as defined in the below author licence), an exclusive licence and/or a non-exclusive licence for contributions from authors who are: i) UK Crown employees; ii) where BMJ has agreed a CC-BY licence shall apply, and/or iii) in accordance with the terms applicable for US Federal Government officers or employees acting as part of their official duties; on a worldwide, perpetual, irrevocable, royalty-free basis to BMJ Publishing Group Ltd ("BMJ") its licensees and where the relevant Journal is co-owned by BMJ to the co-owners of the Journal, to publish the Work in this journal and any other BMJ products and to exploit all rights, as set out in our licence.

The Submitting Author accepts and understands that any supply made under these terms is made by BMJ to the Submitting Author unless you are acting as an employee on behalf of your employer or a postgraduate student of an affiliated institution which is paying any applicable article publishing charge ("APC") for Open Access articles. Where the Submitting Author wishes to make the Work available on an Open Access basis (and intends to pay the relevant APC), the terms of reuse of such Open Access shall be governed by a Creative Commons licence – details of these licences and which Creative Commons licence will apply to this Work are set out in our licence referred to above.

Other than as permitted in any relevant BMJ Author's Self Archiving Policies, I confirm this Work has not been accepted for publication elsewhere, is not being considered for publication elsewhere and does not duplicate material already published. I confirm all authors consent to publication of this Work and authorise the granting of this licence.

For peer review only

Overestimating women's representation in medicine: A survey of medical professionals' estimates, and their (un)willingness to support gender-equality initiatives

Christopher T. Begeny [1*] Rebecca C. Grossman [2] Michelle K. Ryan [1,3]

*corresponding author

[1] Department of Psychology, University of Exeter, United Kingdom
C.Begeny@exeter.ac.uk

[2] Oxford Centre for Diabetes, Endocrinology and Metabolism, University of Oxford, United Kingdom
rebeccagrossman@doctors.org.uk

[3] Global Institute for Women's Leadership, The Australian National University, Australia
Michelle.Ryan@anu.edu.au

Corresponding Author Information
Dr. Christopher T. Begeny
Department of Psychology, University of Exeter
Washington-Singer Laboratories, Perry Road
Exeter, Devon EX4 4QG
United Kingdom

WORD COUNT (incl. abstract, main text, and references): 5,381

Keywords: gender; equality; diversity; medicine; STEMM; stereotypes; bias; women's representation; human resource management, organizational development

For peer review only - http://bmjopen.bmj.com/site/about/guidelines.xhtml

For peer review only - http://bmjopen.bmj.com/site/about/guidelines.xhtml

Abstract

Objective

Amidst growing numbers of women in certain areas of medicine (e.g., general practice/primary care), yet their continued underrepresentation in others (e.g., surgical specialties), this study examines: (i) whether medical professionals mistakenly infer that women are now broadly well represented – overestimating women's *true* representation in several different areas and roles; (ii) whether this overestimation of women's representation predicts decreased support for gender-equality initiatives in the field, in conjunction with one's own gender.

Design

Cross-sectional survey

Setting

UK-based medical field

Participants

425 UK medical consultants/general practitioners and trainees (ST/CT1+/SHO/Registrar); 47% female

Main Outcome Measures

Estimates of women's representation in different areas/roles within medicine, examined as a composite estimate and individually; a multi-item measure of support for gender-based initiatives in medicine

Results

Medical professionals tended to overestimate women's true representation in several different areas of medicine (general practice, medical specialties, surgical specialties) and in various roles (consultants/GPs, trainees, medical school graduates). Moreover, these erroneous estimates predicted a decreased willingness to support gender-based initiatives, particularly among men in the field: composite overestimation*respondent-gender interaction, B = -.04, 95% CI = -.07 to -.01, p = .01. Specifically, while female respondents' (over)estimates were unrelated to their level of support, B = .00, 95% CI = -.02 to .02, p = .92, male respondents' tendency to overestimate the proportion of women in medicine predicted lower support for gender-based initiatives, B = -.04, 95% CI -.06 to -.02, p < .001.

Conclusions

While some progress has been made in gender representation in the medical field, this research illustrates that there are still barriers to gender-equality efforts – and it identifies who within the field is focally maintaining these barriers. It is those individuals (particularly men) who overestimate the *true* progress that has been made in women's representation who are at highest risk for undermining it.

Strengths and Limitations of this Study

1. With women now well represented in some areas of medicine yet underrepresented in others, there remains a dearth of evidence as to whether medical professionals are able to accurately gauge women's representation in different areas/roles; this study is designed to help fill that gap in knowledge.
2. There is also no known evidence as to whether the tendency to overestimate women's true representation can help explain why some medical professionals are reluctant to support gender-equality initiatives in the field; this study is also designed to help fill that gap in knowledge.
3. The design of this research further enables us to help medical professionals and related organizations, as well as policymakers, identify barriers to gender-equality efforts – by identifying who within the field may be most likely to resist or withhold support for initiatives that aim to promote gender equality in the field.
4. More broadly, amidst ongoing efforts to promote gender equality in the medical field, the design of this research allows us to illustrate that it is important not only to consider the true representation of women in the field but also medical professionals' *perceptions* of women's representation.
5. This study was not designed to assess *why* some medical professionals' estimates of women's representation is linked to their level of support for gender-equality initiatives.

Introduction

Paralleling trends in other countries, in the UK women now make up over half of all medical school graduates [1,2]. However, recruitment of female doctors to several specialty areas is not keeping pace with their recruitment to medicine in general [3,4]. For instance, women are well represented in general practice/primary care, yet remain underrepresented in medical and surgical specialties (e.g., in surgical specialties, only 13% of consultants are women) [5].

Despite women's continuing underrepresentation in several areas of medicine (including some of the highest paying and most prestigious areas) [6–8], their more prominent representation in general practice and medical schools may be prompting some in the field to mistakenly infer that women are now well represented across the board, or better represented than they actually are in several areas. This is important to consider, partly because if individuals *overestimate* women's representation they may be less willing to support policies and initiatives that aim to further promote gender equality in the profession. They may regard them as no longer necessary, for instance. Indeed, previous research on this topic, though limited in scope, demonstrates that when individuals overestimate women's representation in a field (e.g., in Science, Technology, Engineering, Mathematics and Medicine [STEMM], politics), they show less support for initiatives that aim to help women in those fields [9–11]. Thus, medical professionals who overestimate the true progress that has been made in women's representation in the field may be at highest risk for undermining it.

Medical professionals' tendency to support gender-equality initiatives may hinge on more than their (over)estimates of women in the field, however. It may also depend on medical professionals' own gender. This is partly because gender-based initiatives and related groups (e.g., the General Medical Council Gender Equality Scheme, Women in Surgery at the Royal College of Surgeons) aim to promote not just the representation of women but also the *equal treatment* of women – a recognition that true gender equality is achieved, and fundamentally defined, not just by numerical representation but the absence of gender bias in how women (and individuals of all genders) are perceived and treated. Thus, representation aside, individuals may continue supporting these gender-based initiatives if they are

cognizant of ongoing issues with gender bias and discrimination in the field [8,12–14]. Indeed, recent evidence demonstrates that even when women become well represented in a field, gender biases and unequal treatment persist, and it is predominantly *women* in the field who remain cognizant of this fact (at significantly higher rates than men) [15]. Ultimately, this suggests women in the medical profession may more reliably support gender-based initiatives, regardless of their estimations of women's numerical representation in the field, because they are more likely to see the ongoing value in these initiatives for combatting gender bias. By comparison, because men are less likely to recognize issues of gender bias, their support for gender-equality initiatives may more simply, and systematically, vary as a function of their tendency to overestimate women's representation.

Current Research

The current research examines whether medical professionals tend to overestimate women's representation in medicine, and whether such erroneous estimates (along with their own gender) predict a decreased willingness to support gender-based initiatives. Using a sample of UK medical professionals, we first test whether individuals are generally accurate in estimating women's representation in different areas of medicine – general practice, medical and surgical specialties – and in different roles – consultants/GPs, trainees/junior doctors, medical school graduates. We then test whether, as hypothesized, overestimating women's representation predicts decreased support for gender-based initiatives, and whether this is moderated by medical professional's own gender.

Gender-Stereotypical Beliefs about Women in Medicine. As an exploratory step, we also examine individuals' endorsement of a gender-stereotypical belief in men's superiority for the medical profession (e.g., that men are simply better suited for the profession) – a belief that implies women should not be afforded equality in the profession, and thus should predict lower willingness to support gender-equality initiatives [16,17]. Thus, assessing this belief offers two potential insights. First, it allows us to test our core hypothesis – that overestimating women's representation predicts less support for gender-based initiatives, primarily among men – more conservatively, by testing whether this effect (overestimation*respondent-gender interaction) is robust even when accounting for the role of this belief

in explaining individuals' (lacking) support for gender-based initiatives. Second, it allows us to assess whether there might be some men, like some women in medicine, who overestimate women's representation yet maintain a consistent level of support for these initiatives. This may be the case among men who more strongly reject this belief (tested via an overestimation*respondent-gender*gender-stereotypical belief interaction).

Methods

Participants and Procedure

Participants were 425 UK-based consultants/general practitioners (GPs) and trainees/junior doctors (grades: ST/CT1+/SHO/Registrar) in the medical field (47% female; M_{age}=42.63, SD=11.82; role: 13.9/4.5% consultants/trainees in general practice, 24.6/12.0% consultants/trainees in medicine, 7.9/6.7% consultants/trainees in surgery, 7.4% foundation year 1/2 doctors, 23.0% other (e.g., doctors in industry positions, doctors in psychiatry). For more detailed descriptions of these areas and roles within medicine, see [18,19]. Respondents completed a brief survey online described as aiming to "better understand individuals' perceptions of doctors within the UK medical profession." We recruited participants via email, disseminated through list-servs maintained by the 24 medical Royal Colleges and Faculties, 214 NHS Trusts, and 46 medical sub-specialty and social societies. We also recruited respondents via social media and a doctors-only web forum. Participation was voluntary (no remuneration). We excluded four respondents because they indicated that they did not work (nor had worked) in the UK, and three for illogical responses (stating that they believed 98-100% of all consultants and trainees, across all areas, were female; final sample size, n = 418; ; n = 377-418 for all primary analyses [missing data: 0-25 cases for area/role-specific estimates of women's representation, 41 cases for measure of support for gender-based initiatives]). Sensitivity analyses indicated sample size was generally adequate (based on lowest n, α=.05, 1-β=.80; for detecting $d \geq .14$ in one-sample t tests, for detecting $f^2 \geq .02$ based on ΔR^2 for the addition of the overestimation*respondent-gender interaction term). All data underlying the findings described in this article are available at The Center for Open Science [20].

Patient and Public Involvement

No patient involved; neither patients nor the public were directly involved in the design, conduct, reporting, or dissemination plans of this research.

Measures

Respondents answered questions measuring the following key constructs, and provided demographic information (e.g., gender, age, general area/role in medicine).

Estimates of Females by Area/Role. To assess respondents' estimates of the proportions of women in different areas/roles, we asked, "What percentage of ___ do you think are female?" with the following inserted: GP (general practitioner) doctors, trainee GP (general practitioner) doctors (ST/CT1+/SHO/Registrar), consultant doctors in medical specialties, trainee doctors in medical specialties (ST/CT1+/SHO/Registrar), consultant doctors in surgical specialties, trainee doctors in surgical specialties (ST/CT1+/SHO/Registrar), medical school graduates. Respondents answered each of these seven questions on a sliding scale from 0-100%. To calculate the degree to which participants under- or overestimated true proportions, we subtracted the actual proportion of females within each area/role (obtained statistics aligned to the time of data collection 2017; [21,22]) from respondents' estimate. Thus, positive values reflected overestimation.

Support for Gender-based Initiatives in the Profession. To assess support for initiatives designed to support women in the UK medical profession, after explaining that such initiatives exist and providing examples (e.g., the General Medical Council Gender Equality Scheme, Women in Surgery at the Royal College of Surgeons) we asked respondents to indicate how much they (dis)agree that these types of initiatives are: necessary, fair, excessive/'over the top' (reverse scored), or put men at a disadvantage (reverse scored). These four items were rated 1-7 (*Strongly Disagree* - *Strongly Agree*), reliable ($\alpha = .85$), and averaged to form a composite.

Gender-Stereotypical Beliefs about Women in Medicine. To assess endorsement of a gender-stereotypical belief about men's superiority for the medical profession, we asked respondents how much they (dis)agree that, e.g., there is something about being a man that makes one better suited for the medical profession (adapted; [16]). These six items were rated 1-7 (*Strongly Disagree* - *Strongly Agree*),

reliable (α = .80), and averaged to form a composite.

Overview of Statistical Methods

All statistical analyses were conducted in SPSS (pairwise deletion used as necessary). This included bivariate (zero-order, Pearson) correlations (see Table 1), one-sample *t*-tests (see Tables 2 and 3; test value = 0), independent samples *t*-tests (see Table 3 superscripts), and tests of interactions using linear (ordinary least squares) regression via the PROCESS macro in SPSS, with 5,000 resamples for generating percentile bootstrap confidence intervals (for more details about PROCESS, see [23]). Primary regression analyses tested whether respondents' support for gender-based initiatives varied as function of their tendency to overestimate the proportion of women in medicine and their own gender (overestimation*respondent-gender interaction) using PROCESS Model 1 (outcome: support for gender-based initiatives; predictor: overestimation of women's representation [mean-centered]; moderator: gender [0 *female*, 1 *male*; mean-centered]; covariate: age; analyses without covariate evinced the same statistically significant results). Follow-up regression analyses mirrored primary regression analyses while further testing whether the hypothesized overestimation*respondent-gender effect was robust and/or qualified by respondents' endorsement of the gender-stereotypical belief that men are superior for the medical profession (overestimation*respondent gender*gender-stereotypical belief) using PROCESS Model 3 (regression model identical to the primary regression model, but with the inclusion of a second moderator, endorsement of gender-stereotypical belief, and its corresponding interaction terms).

Results

Table 1 provides bivariate correlations illustrating how female and male medical professionals' tendency to overestimate women's representation in a given area/role correspond to their overestimations in other areas/roles, as well as their endorsement of gender stereotypical beliefs and support for gender-based initiatives.

Table 1. Bivariate (zero-order) correlations by gender, with correlations among female and male respondents above and below the diagonal respectively.

	(above/below diagonal: correlations within female/male respondents, respectively)								
Variable	1	2	3	4	5	6	7	8	9
(Over)estimated % of female:									
1. TRs, general practice	----	.48***	.39***	.49***	.30***	.19**	.61***	.13^{+}	.11
2. TRs, medicine	.51***	----	.39***	.26***	.42***	.26***	.43***	.07	.05
3. TRs, surgery	.20**	27***	----	.32***	.41***	.55***	.45***	.10	-.04
4. DRs, general practice	.64***	48***	.11^{+}	----	.40***	.12^{+}	.33***	.14^{+}	.05
5. DRs, medicine	.21**	.45***	.30***	38***	----	.53***	.35***	.04	-.05
6. DRs, surgery	.16*	27***	.46***	25***	.52***	----	.19**	.05	-.17*
7. Med. school graduates	.61***	48***	.09	53***	.18**	.15*	----	.04	.13^{+}
8. Gender Stereotypical Beliefs	.07	.11	.00	.04	.05	.08	.05	----	-.28***
9. Support for Gender Initiatives	-.15*	-.17*	-.06	-.14^{+}	-.16*	-.18**	-.15*	-.57***	----

TRs = Trainee/junior doctors (ST/CT1+/SHO/Registrar), DRs = GP/Consultant doctors; the numbering across the top row of the table (1-9) correspond to the variables, as numbered, in the left column.
$^{***}p \leq .001$, $^{**}p \leq .01$, $^{*}p \leq .05$ $^{+}p \leq .10$

Respondent Estimates versus Actual Proportions of Women by Area/Role

We first examined how respondents' estimated proportions of women in different areas/roles compared to actual proportions. Across areas, both male and female respondents tended to overestimate the proportion of female consultants and GPs. Estimated proportions of female trainees varied more by area. As noted in Table 2, these results were also largely evident (among both male and female respondents) when limiting analyses for a given area to the respondents who were themselves in that particular area of medicine. Results also showed that both male and female respondents overestimated the proportion of female medical school graduates. See Table 3 for results separated by respondent gender.

Tables 2 and 3 also show the standard deviations for each mean estimated proportion. These highlight that, irrespective of the estimated proportion of women in an area/role *on average*, there was

substantial variability in estimates within the sample of respondents. This variability is key to assessing whether these (over)estimations reliably predict individuals' (lower) levels of support for gender-based initiatives.

Table 2. Respondent estimates versus actual proportions of women by area/role.

**Role**	**Area**	**Estimated % Female (SD)**		**Actual % Female**	**Difference (Est. - Actual)**				
Consultants/GPs	General Practice	58.25	(11.49)	54	4.25	[3.15 to 5.36]	$t = 7.57$	$p < .001$ [a]	$d = .37$
	Medicine	43.27	(11.15)	37	6.27	[5.20 to 7.34]	$t = 11.50$	$p < .001$ [a]	$d = .56$
	Surgery	24.99	(10.65)	14	10.99	[9.97 to 12.02]	$t = 21.10$	$p < .001$ [a]	$d = 1.03$
Trainees	General Practice	63.55	(12.35)	69	- 5.45	[-6.68 to -4.23]	$t = -8.75$	$p < .001$	$d = .44$
	Medicine	53.82	(10.15)	53	0.82	[-0.19 to 1.83]	$t = 1.60$	$p = .11$ [a]	$d = .08$
	Surgery	37.37	(11.91)	33	4.37	[3.19 to 5.55]	$t = 7.27$	$p < .001$ [a]	$d = .37$
Medical School Graduates		59.68	(9.83)	55	4.68	[3.70 to 5.65]	$t = 9.44$	$p < .001$ [a]	$d = .48$

Positive difference scores indicate overestimations of women's representation; values in brackets are 95% confidence intervals around that difference score; t, p, and d values indicate whether that difference score deviated significantly from zero (one-sample t-test, effect size d; i.e., whether estimations of women's representation significantly differed from their true representation); [a] Virtually identical results evident (for both male and female respondents) when limiting analyses to respondents (trainees and consultants/GPs) who were themselves in this area of medicine (analyses not applicable regarding medical school graduates). Actual percentages reflect statistics aligned to the time of data collection (obtained from [21,22]).

For peer review only - http://bmjopen.bmj.com/site/about/guidelines.xhtml

Table 3. Respondent estimates versus actual proportions of women by area/role, examined separately for male and female respondents.

Role	Area	Estimated % Female (SD)				Actual % Female	Difference (Est. - Actual)		
Consultants/GPs	General Practice	*Est. by*	*Male Respondents*	56.83	(11.14)	54	2.83 [a]	[1.35 to 4.31]	$t = 3.77$
			Female Respondents	59.83	(11.69)		5.83 [a]	[4.19 to 7.47]	$t = 7.01$
	Medicine	*Est. by*	*Male Respondents*	42.76	(10.61)	37	5.76	[4.35 to 7.17]	$t = 8.06$
			Female Respondents	43.83	(11.72)		6.83	[5.19 to 8.48]	$t = 8.21$
	Surgery	*Est. by*	*Male Respondents*	24.75	(10.62)	14	10.75	[9.34 to 12.17]	$t = 15.02$
			Female Respondents	25.26	(10.71)		11.26	[9.76 to 12.76]	$t = 14.79$
Trainees	General Practice	*Est. by*	*Male Respondents*	62.28	(11.91)	69	-6.72 [b]	[-8.36 to -5.08]	$t = -8.08$
			Female Respondents	64.93	(12.70)		-4.07 [b]	[-5.90 to -2.24]	$t = -4.39$
	Medicine	*Est. by*	*Male Respondents*	53.15	(10.28)	53	0.15	[-1.27 to 1.56]	$t = 0.20$
			Female Respondents	54.55	(9.99)		1.55	[0.12 to 2.99]	$t = 2.13$
	Surgery	*Est. by*	*Male Respondents*	37.36	(11.48)	33	4.36	[2.78 to 5.94]	$t = 5.43$
			Female Respondents	37.38	(12.40)		4.38	[2.59 to 6.16]	$t = 4.84$
Medical School Graduates		*Est. by*	*Male Respondents*	59.75	(8.48)	55	4.75	[3.58 to 5.92]	$t = 8.02$
			Female Respondents	59.60	(11.13)		4.60	[2.99 to 6.20]	$t = 5.66$

Positive difference scores indicate overestimations of women's representation; values in brackets are 95% confidence intervals around that difference score; t, p, and d values indicate whether that difference score deviated significantly from zero (one-sample t-test, effect size d; i.e., whether estimations of women's representation significantly differed from their true representation); [a/b] The magnitude of male and female respondents' over/underestimations (i.e., their mean deviations from the actual % female) for this area/role significantly differed from one another (t's = 2.68/2.14, p's = .01/.03, d's = .26/.22). For all other areas/roles (without a superscript), male and female respondents' overestimations did not significantly differ from one another (all t's ≤ 1.37, p's ≥ .17). Actual percentages reflect statistics aligned to the time of data collection (obtained from [21,22]).

Support for Gender-based Initiatives

To test whether respondents' support for gender-based initiatives varied by their tendency to overestimate the proportion of women in medicine and their own gender, we ran tests of interactions via PROCESS (Model 1; see Overview of Statistical Methods for more detail). Given that the measure of support for gender-based initiatives was not tied to one specific area or role within medicine, it is

For peer review only - http://bmjopen.bmj.com/site/about/guidelines.xhtml

arguably most relevant to assess how respondents' levels of support varied as a function of their *overall* tendency to overestimate women's representation (aggregated across areas/roles). We therefore computed a composite score (M=3.84, SD=7.47) reflecting respondents' average tendency to overestimate women's representation across the seven aforementioned areas/roles (α=.80 for the seven estimated areas/roles).

As Figure 1 shows, results evinced differences in support for gender-based initiatives as a function of respondents' tendency to overestimate the proportion of women in medicine and their own gender (overestimation*respondent-gender interaction, B=-.04, 95% CI=-.07 to -.01, p=.01, ΔR^2=.02 for the addition of interaction term, $F(1,372)$=6.48, p=.01, f^2=.02; overall $F(4,372)$=8.53, $p < .001$; overestimation, B=-.02, 95% CI=-.04 to -.01, p=.01; respondent gender, B=-.40, 95% CI=-.65 to -.16, p=.001). Generally speaking, this means that as medical professionals got more severe in their overestimations of women's true representation, the disparity between female and male medical professionals' support for gender-based initiatives grew larger – as illustrated in Figure 1.

Tests of simple slopes further showed that female respondents' (over)estimates were unrelated to their level of support (B=.00, 95% CI=-.02 to .02, p=.92), yet male respondents' tendency to overestimate the proportion of women in medicine predicted lower support for gender-based initiatives (B=-.04, 95% CI=-.06 to -.02, $p < .001$). In other words, among female respondents, regardless of their estimations of women in medicine, there was no systematic difference in their level of support for gender-based initiatives. Yet among male respondents, there were systematic differences; in essence, for every 1% increase in their (over)estimations of the proportion of women in medicine, men's support for gender-based initiatives dropped by .04 points on average (thus, being 12% higher in one's overestimations equated to approximately a half-point decrease in level of support; see Figure 1 for a visual illustration).

For peer review only - http://bmjopen.bmj.com/site/about/guidelines.xhtml

Figure 1. Male and female respondents' (i.e., medical professionals') support for gender-based initiatives in the UK medical profession (1-7 scale), as a function of their estimates of the proportion of women in medicine. Positive values on the x-axis reflect an overestimation of women's representation. Female respondents' estimates were unrelated to their level of support (B = .00, 95% CI = -.02 to .02, p = .92). By comparison, male respondents' tendency to overestimate the proportion of women in medicine predicted significantly less support for gender-based initiatives (B = -.04, 95% CI = -.06 to -.02, p < .001; overestimation*respondent-gender interaction, B = -.04, 95% CI = -.07 to -.01, p = .01, ΔR^2 = .02 for the addition of interaction term, $F(1,372)$ = 6.48, p = .01, f^2 = .02).

We also tested these interaction effects by area/role. As Figure 2 shows, regarding estimates of female trainees in general practice, results showed the same pattern of results (overestimation*respondent-gender interaction, B=-.03, 95% CI=-.05 to -.01, p=.01, ΔR^2=.02 for addition of interaction term, $F(1,372)$=7.13, p=.01; overall $F(4,372)$=7.37, p < .001). Simple slopes showed that female respondents' estimates of female trainees in this area were unrelated to their level of support (B=.01, 95% CI=-.01 to .02, p=.30), yet male respondents' tendency to overestimate the proportion of women in this area predicted less support for gender-based initiatives (B=-.02, 95% CI=-.03 to -.01, p=.01). This same pattern was also found regarding estimates of female trainees in medicine (overestimation*respondent-gender interaction, B=-.03, 95% CI=-.05 to -.003, p=.03; simple slopes:

female respondents, *B*=.00, 95% CI=-.01 to .02, *p*=.71; male respondents *B*=-.02, , 95% CI=-.04 to -.01, *p*=.01), though not for surgery where, notably, women's representation is still quite low (overestimation*respondent-gender interaction, *B*=.00, 95% CI=-.02 to .02, *p*=.65). Regarding estimates of female medical school graduates, results again evinced a significant interaction (overestimation*respondent-gender interaction, *B*=-.03, 95% CI=-.07 to -.01, *p*=.01; simple slopes: female respondents, *B*=.01, 95% CI=-.01 to .02, *p*=.22; male respondents *B*=-.02, 95% CI=-.04 to -.003, *p*=.02).

Figure 2. Male and female respondents' (i.e., medical professionals') support for gender-based initiatives in the UK medical profession (1-7 scale), as a function of their estimates of the proportion of: (i) female trainees in general practice, (ii) medicine, and (iii) surgery, and (iv) female medical school graduates. Positive values on the x-axis reflect an overestimation of women's representation in that area/role. In the areas of general practice and medicine, and regarding medical school graduates, female respondents' estimates were unrelated to their level of support, yet male respondents' tendency to overestimate the representation of women in these areas/roles predicted significantly less support for gender-based initiatives. In surgery, neither women's nor men's estimates of female trainees predicted level of support.

For peer review only - http://bmjopen.bmj.com/site/about/guidelines.xhtml

This same pattern of results was also evident when examining respondents' estimates of female GPs/consultants by area, though effects were more modest (overestimation*respondent-gender interactions: general practice, *B*=-.02, 95% CI=-.04 to .00, *p*=.06; medicine, *B*=-.01, 95% CI=-.03 to .01, *p*=.17; surgery, *B*=-.01, 95% CI=-.03 to .02, *p*=.61). Again, in areas of general practice and medicine (not surgery), female respondents' estimates of female doctors in these areas were unrelated to their level of support (simple slopes for female respondents: general practice, *B*=.00, 95% CI=-.01 to .02, *p*=.81, medicine, *B*=-.01, 95% CI=-.02 to .01, *p*=.35, surgery, *B*=-.02, 95% CI=-.03 to .00, *p*=.05). Yet male respondents' tendency to overestimate the proportion of female doctors in these areas predicted less support for gender-based initiatives (simple slopes for male respondents: general practice, *B*=-.02, 95% CI=-.03 to -.004, *p*=.01, medicine, *B*=-.02, 95% CI=-.04 to -.01, *p*=.01, surgery, *B*=-.02, 95% CI=-.04 to -.01, *p*=.004).

Follow-up Analysis. In follow-up analysis (PROCESS Model 3; paralleling primary analysis using overestimation composite), we tested whether the hypothesized overestimation*respondent-gender effect was robust and/or qualified by respondents' endorsement of the gender-stereotypical belief that men are superior for the medical profession.

Results showed that those who more strongly endorsed this belief had less support for gender-based initiatives (gender-stereotypical belief: *B*=-.44, 95% CI=-.53 to -.34, $p < .001$; overestimation, *B*=-.01, 95% CI=-.03 to .00, *p*=.06; respondent gender, *B*=-.34, 95% CI=-.55 to -.13, *p*=.001; overall $F(8,362)=18.90$, $p < .001$). Yet at the same time, the hypothesized overestimation*respondent-gender interaction remained significant (*B*=-.04, 95% CI=-.07 to -.01, *p*=.01). Thus, even when accounting for the role of individuals' endorsement of this belief, their level of support for gender-based initiatives still systematically varied by the tendency to overestimate the proportion of women in medicine and their own gender. Results also showed that this interaction was not qualified by a three-way interaction (overestimation*respondent gender*gender-stereotypical belief; *B*=-.01, 95% CI=-.03 to .02, *p*=.70), further illustrating its robustness in explaining individuals' support for gender-based initiatives.

While the three-way interaction was nonsignificant, the hypothesized effect at different levels of

For peer review only - http://bmjopen.bmj.com/site/about/guidelines.xhtml

endorsement of this gender-stereotypical belief did illustrate a potentially informative pattern of results. Specifically, male and female respondents who overestimated the proportion of women in medicine but also strongly *rejected* this belief (at the 25th percentile in the belief-endorsement range) did not differ in their level of support for gender-based initiatives (B=-.03, 95% CI=-.07 to .01, p=.14): neither female (B=.01, 95% CI=-.02 to .04, p=.62) nor male (B=-.02, 95% CI=-.05 to .01, p=.11) respondents' tendency to overestimate the proportion of women in medicine predicted less support for initiatives. Yet among those who more strongly *endorsed* this belief (at the 75th percentile), male and female respondents did differ in their support (B=-.04, 95% CI=-.07 to -.01, p =.01): female respondents' overestimates were unrelated to support (B=.00, 95% CI=-.02 to .03, p=.78) while male respondents' overestimates predicted less support for gender-based initiatives (B=-.04, 95% CI=-.06 to -.02, p=.001), such that among male respondents who more strongly endorsed this belief, every 1% increase in their (over)estimations of women in medicine equated to an average .04 point drop in support for gender-based initiatives. Thus, while these analyses were exploratory, they suggest that men who overestimate women's representation may not be invariably more reluctant to support gender-based initiatives. There may be a subset of men who, despite overestimating women's representation, maintain a level of support for gender-based initiatives on par with that of their female counterparts – specifically, those men who more strongly reject the gender-stereotypical belief that men are more suitable for the profession.

Discussion

The strength and quality of the medical profession – including its ability to address an array of public health issues, and to ensure patient satisfaction – hinges on recruiting, retaining and supporting the full range of diverse talent that exists in the population, including among women [14,24]. In this vein, various initiatives are underway to increase women's representation in medicine, with some signs of progress.

Yet amidst this growing gender diversity in medicine – with women now well represented in some areas, yet underrepresented in others – it is important to understand how medical professionals are perceiving this changing demographic landscape. The current research shows that amidst growing

For peer review only - http://bmjopen.bmj.com/site/about/guidelines.xhtml

numbers of women, medical professionals are tending to overestimate women's true representation, with adverse implications. This research shows that when individuals – particularly men – overestimate the proportion of women in medicine they express less support for gender-based initiatives that are striving to promote greater equality. Thus, men who overestimate the *true* progress that has been made in women's representation are at highest risk for undermining it.

This points to an insidious consequence that can arise when women's representation grows within a given field. It seems to prompt some to misperceive and overstate the actual degree of change, and following from this, particularly for men, mistakenly infer that gender-equality initiatives in the field are no longer worth supporting. This ultimately hinders efforts to promote true equality – whether it be promoting women's representation in areas of the field where they are still underrepresented, or combatting issues of gender bias that exist independent of women's numerical representation [15].

In practical terms, this research illustrates the very real nature of the issue – that medical professionals are indeed overestimating women's representation in several areas and roles in the field. Simultaneously, it helps identify *who* within the field is at highest risk for resisting efforts to promote gender equality.

This study does have its limitations. These include uncertainty around the total number of medical professionals who saw the study invitation (given methods for dissemination) and thus the response rate. Additionally, while this study examined estimates of women's representation across seven different key areas and roles, including GPs/consultants and trainees, future research might examine additional roles (e.g., Specialty and Associate Specialist doctors) or specialty areas.

The cross-sectional nature of these data precludes tests of causality. However, previous experimental work supports our hypothesized directionality of effect [16], suggesting that when (male) medical professionals overestimate growth in the number of women in their field it results in less support for gender-based initiatives.

In future research, it will also be important to probe the mechanisms underpinning this overestimation effect. One possibility is that overestimating women's representation prompts individuals,

For peer review only - http://bmjopen.bmj.com/site/about/guidelines.xhtml

particularly men, to genuinely albeit naïvely infer that gender bias is no longer an issue in their profession – that the biases and discrimination that once prevented women from entering the field are no longer occurring (see also [9,15]). As a result, they may regard ongoing gender-based initiatives as unnecessary.

Another possibility is that overestimating women's representation predicts lower support for gender-based initiatives because that overestimation reflects a heightened sense of threat that some men feel, prompting them to exert more resistance to that changing demographic landscape (e.g., expressing less support for gender-based initiatives) [16]. Notably though, our overestimation*respondent-gender effect held true when accounting for individuals' endorsement of the gender-stereotypical belief that men are better suited for the medical profession. This is important because research suggests endorsement of such a belief *reflects* men's sense of threat (i.e., they endorse this type of belief when they feel their high status position in a profession is threatened) [17]. In this way, it seems that an overestimation effect may stand independent of, or is at least not fully explained by, a sense of threat induced by a perceptible growth in women in the field.

Overall, this suggests multiple strategies may be required to address the consequences of this overestimation effect, depending on whether or for whom it is underpinned by a sense of threat versus naïveté about ongoing issues of underrepresentation (if not also ongoing issues of gender bias). For instance, targeted information campaigns that increase knowledge and awareness about women's true representation in different areas of medicine – along with information about persisting forms of gender bias (separate from matters of representation) – may be useful in fostering greater support for gender-based initiatives among medical professionals whose reservations about these initiatives are rooted in genuine naïveté about persisting issues with underrepresentation and bias. Yet among those whose resistance is rooted in a sense of threat by growing proportions of women in the profession, other strategies may be necessary (e.g., work-related self-affirmation techniques that alleviate this sense of threat) [25,26]. There are a number of other potential strategies to consider as well, including those that aim to directly promote greater gender equality (for reviews, see [14,27]).

It will also be important to consider whether there are thresholds for spurring this effect. In the

For peer review only - http://bmjopen.bmj.com/site/about/guidelines.xhtml

current research, we found that while overestimations of women across most areas/roles predicted lower support for gender-equality initiatives, this was not so for surgical specialties (both regarding estimates of GPs/consultants and trainees). This may be because both the actual representation and individuals' overestimations of women in this area are still relatively low (e.g., actual and estimated proportions of female consultants in surgery: 14% and 25%; see Table 2). This suggests that when it is still quite clear that women are vastly underrepresented, aversion to gender-equality initiatives is not piqued – perhaps either because it remains clear that those initiatives are still necessary (from the perspective of a "naïve" over-estimator), or because the still-low representation of women does not yet elicit threat (from the perspective of a "threatened" over-estimator).

It is also notable that medical professionals' endorsement of the gender-stereotypical belief that men are better suited for the profession was unrelated to their tendency to overestimate the proportion of women in the field (see Table 1). This held true for both male and female respondents. It suggests that overestimations of women's representation do not simply reflect a negative, pre-existing attitude (about women's suitability for the profession). Thus, while future research should further probe this relationship, their independence here indicates that medical professionals' estimates of women's representation are, in their own right, an important basis for understanding who is likely to support gender-equality initiatives, or resist them – particularly among men in the profession. While endorsement of this gender-stereotypical belief is important to consider, medical professionals' (over)estimations of women is key too.

Going forward, it will also be important to probe the role of gender in moderating the evinced overestimation effect. One possibility is that this gender-moderated effect reflects the fact that men are more likely than women to be unaware of – or simply deny – that gender bias is still an issue in their profession (i.e., in the most precise theoretical terms, it is one's belief that gender bias is no longer an issue, more than gender, that moderates the effect; [15,28]). Another possibility is that this gender-moderated effect reflects an expression of ingroup favoritism [29,30]; if individuals perceive gender-based initiatives as generally beneficial to women (as a group) but not men, and they are motivated to act in ways that support their own gender-based ingroup (e.g., if they highly identify with their gender),

For peer review only - http://bmjopen.bmj.com/site/about/guidelines.xhtml

women may be generally supportive of these initiatives while men may not be, especially if men's overestimation of women in the field helps justify a belief that making deliberate efforts to support members of an outgroup are no longer necessary (i.e., supporting initiatives that perceptibly benefit women).

Future research might also examine whether the general public similarly tends to overestimate women's representation in the medical profession. Individuals outside the profession would presumably be just as prone, if not more so, to these erroneous estimates. If so, given the current evidence that this has adverse implications for one's willingness to support gender-equality initiatives, this would underscore the gravity of the issue – highlighting that resistance to establishing gender equality in the medical field may be coming from both those within and outside of the profession. In a similar vein, it will be valuable to examine whether these processes are evident specifically among leaders within the medical profession.

Conclusion

Amidst ongoing efforts to promote greater gender equality in medicine, the current research illustrates that it is important not only to consider the true representation of women in the field, but also medical professionals' *perceptions* of women's representation. As shown, individuals' (mis)perceptions are accompanied by growing reservations, or less support for, gender-equality initiatives. In this way, individuals' erroneous estimates mean less support for initiatives that are ultimately working to make the profession *truly* equitable for women.

For peer review only - http://bmjopen.bmj.com/site/about/guidelines.xhtml

References

1 GMC. The state of medical education and practice in the UK: The workforce report. General Medical Council 2019. https://www.gmc-uk.org/about/what-we-do-and-why/data-and-research/the-state-of-medical-education-and-practice-in-the-uk/workforce-report-2019 (accessed 16 Mar 2021).

2 AAMC. The Majority of U.S. Medical Students Are Women, New Data Show. Association of American Medical Colleges 2019. https://www.aamc.org/news-insights/press-releases/majority-us-medical-students-are-women-new-data-show (accessed 16 Mar 2021).

3 RCP. 2016–17 census: UK consultants and higher specialty trainees. Royal College of Physicians 2017. https://www.rcplondon.ac.uk/projects/outputs/2016-17-census-uk-consultants-and-higher-specialty-trainees

4 AAMC. 2020 Physician Specialty Data Report. Association of American Medical Colleges 2020. https://www.aamc.org/data-reports/workforce/interactive-data/active-physicians-sex-and-specialty-2019

5 RCS. Royal College of Surgeons of England, Statistics: Women in Surgery. 2020.https://www.rcseng.ac.uk/careers-in-surgery/women-in-surgery/statistics/

6 Creed PA, Searle J, Rogers ME. Medical specialty prestige and lifestyle preferences for medical students. *Soc Sci Med* 2010;**71**:1084–8. doi:10.1016/j.socscimed.2010.06.027

7 Album D, Westin S. Do diseases have a prestige hierarchy? A survey among physicians and medical students. *Soc Sci Med* 2008;**66**:182–8. doi:10.1016/j.socscimed.2007.07.003

8 Dacre J, Woodhams C. Mend the Gap: The Independent Review into Gender Pay Gaps in Medicine in England. UK Department of Health & Social Care 2020. https://assets.publishing.service.gov.uk/government/uploads/system/uploads/attachment_data/file/944246/Gender_pay_gap_in_medicine_review.pdf

9 Swim JK, Aikin KJ, Hall WS, *et al.* Sexism and racism: Old-fashioned and modern prejudices. *J Pers Soc Psychol* 1995;**68**:199–214. doi:10.1037/0022-3514.68.2.199

10 Sanbonmatsu K. Gender-Related Political Knowledge and the Descriptive Representation of Women. *Polit Behav* 2003;**25**:367–88. doi:10.1023/B:POBE.0000004063.83917.2d

11 Coffé H, Reiser M. How perceptions and information about women's descriptive representation affect support for positive action measures. *Int Polit Sci Rev* 2021;:0192512121995748. doi:10.1177/0192512121995748

12 Burns KEA, Straus SE, Liu K, *et al.* Gender differences in grant and personnel award funding rates at the Canadian Institutes of Health Research based on research content area: A retrospective analysis. *PLOS Med* 2019;**16**:e1002935. doi:10.1371/journal.pmed.1002935

13 Jagsi R, Griffith KA, Stewart A, *et al.* Gender Differences in the Salaries of Physician Researchers. *JAMA* 2012;**307**. doi:10.1001/jama.2012.6183

14 National Academies of Sciences, Engineering, and Medicine. Promising Practices for Addressing the Underrepresentation of Women in Science, Engineering, and Medicine: Opening Doors. Washington,

For peer review only - http://bmjopen.bmj.com/site/about/guidelines.xhtml

DC: : The National Academies Press 2020. https://www.nap.edu/catalog/25585/promising-practices-for-addressing-the-underrepresentation-of-women-in-science-engineering-and-medicine (accessed 1 Mar 2020).

15 Begeny CT, Ryan MK, Moss-Racusin CA, *et al.* In some professions, women have become well represented, yet gender bias persists—Perpetuated by those who think it is not happening. *Sci Adv* 2020;**6**:eaba7814. doi:10.1126/sciadv.aba7814

16 Danbold F, Huo YJ. Men's defense of their prototypicality undermines the success of women in STEM initiatives. *J Exp Soc Psychol* 2017;**72**:57–66. doi:10.1016/j.jesp.2016.12.014

17 Morton TA, Postmes T, Haslam SA, *et al.* Theorizing gender in the face of social change: Is there anything essential about essentialism? *J Pers Soc Psychol* 2009;**96**:653–64. doi:10.1037/a0012966

18 BMA. Doctors' titles explained. Br. Med. Assoc. 2021.https://www.bma.org.uk/advice-and-support/international-doctors/life-and-work-in-the-uk/toolkit-for-doctors-new-to-the-uk/doctors-titles-explained (accessed 4 Jan 2022).

19 NHS. Roles for doctors. Health Careers. 2020.https://www.healthcareers.nhs.uk/explore-roles/doctors/roles-doctors (accessed 4 Jan 2022).

[dataset] 20 Begeny CT, Grossman RC, Ryan MK. Data from: Overestimating women's representation in medicine: A survey of medical professionals' estimates, and their (un)willingness to support gender-equality initiatives. Open Science Framework, June 22, 2021. https://osf.io/hrm63/

21 GMC. The state of medical education and practice in the UK. General Medical Council 2017. https://www.gmc-uk.org/static/documents/content/SoMEP-2017-final-executive-summary.pdf (accessed 16 Mar 2021).

22 NHS Digital. UK National Health Service, HCHS doctors by speciality, grade and gender: April 2015-April 2018. 2018.https://digital.nhs.uk/data-and-information/find-data-and-publications/supplementary-information/2018-supplementary-information-files/staff-numbers/consultants-and-doctors/hchs-doctors-by-speciality.-grade-and-gender-april-2015--april-2018

23 Hayes AF. *Introduction to mediation, moderation, and conditional process analysis: A regression-based approach*. New York, NY, US: : Guilford Press 2013.

24 Grossman RC. The Gender Pay Gap in Medicine: Causes and Solutions. In: Bellini MI, Papalois VE, eds. *Gender Equity in the Medical Profession*. Hershey, PA: : IGI Global 2020. 110–27.http://doi:10.4018/978-1-5225-9599-1.ch008 (accessed 16 Mar 2021).

25 Scheepers D, Ellemers N. Status Stress: Explaining Defensiveness to the Resolution of Social Inequality in Members of Dominant Groups. In: Jetten J, Peters K, eds. *The Social Psychology of Inequality*. Cham: : Springer International Publishing 2019. 267–87. doi:10.1007/978-3-030-28856-3_17

26 Wilkins CL, Kaiser CR. Racial Progress as Threat to the Status Hierarchy: Implications for Perceptions of Anti-White Bias. *Psychol Sci* 2014;**25**:439–46. doi:10.1177/0956797613508412

For peer review only - http://bmjopen.bmj.com/site/about/guidelines.xhtml

27 Begeny CT, Wong CYE, Kirby TA, *et al.* Gender, race, and leadership. Oxf. Res. Encycl. Psychol. 2021. doi:10.1093/acrefore/9780190236557.013.450

28 Moss-Racusin CA, Dovidio JF, Brescoll VL, *et al.* Science faculty's subtle gender biases favor male students. *Proc Natl Acad Sci* 2012;**109**:16474–9. doi:10.1073/pnas.1211286109

29 Brewer MB. The Psychology of Prejudice: Ingroup Love and Outgroup Hate? *J Soc Issues* 1999;**55**:429–44. doi:10.1111/0022-4537.00126

30 Tajfel H, Turner JC. An integrative theory of intergroup conflict. In W. G. Austin & S. Worchel (Eds.). In: *The social psychology of intergroup relations*. Monterey, CA: : Brooks/Cole 1979. 33–47.

For peer review only - http://bmjopen.bmj.com/site/about/guidelines.xhtml

Author Contributions

Conceptualization: CTB, RCG, MKR

Data Curation: CTB, RCG

Formal Analysis: CTB

Funding Acquisition: MKR

Methodology & Design: CTB, RCG, MKR

Project Management & Administration: CTB

Visualization: CTB

Writing, original draft: CTB, RCG

Writing, review & editing: CTB, RCG, MKR

Funding

This work was supported by a European Research Council Consolidator Grant (ERC-CoG 725128) awarded to MKR.

Competing Interests

The authors declare no competing interests. The funders had no role in study design, data collection, analysis or interpretation, nor in the preparation or decision to submit this work for publication.

Ethics Approval

This research was approved by and carried out in compliance with standards for human research set forth by the University of Exeter Ethics Committee (approval for eCLESPsy000134). Informed consent was obtained from participants.

Data Sharing Statement

All data underlying the findings described in this article are available at The Center for Open Science (https://osf.io/hrm63/).

For peer review only - http://bmjopen.bmj.com/site/about/guidelines.xhtml

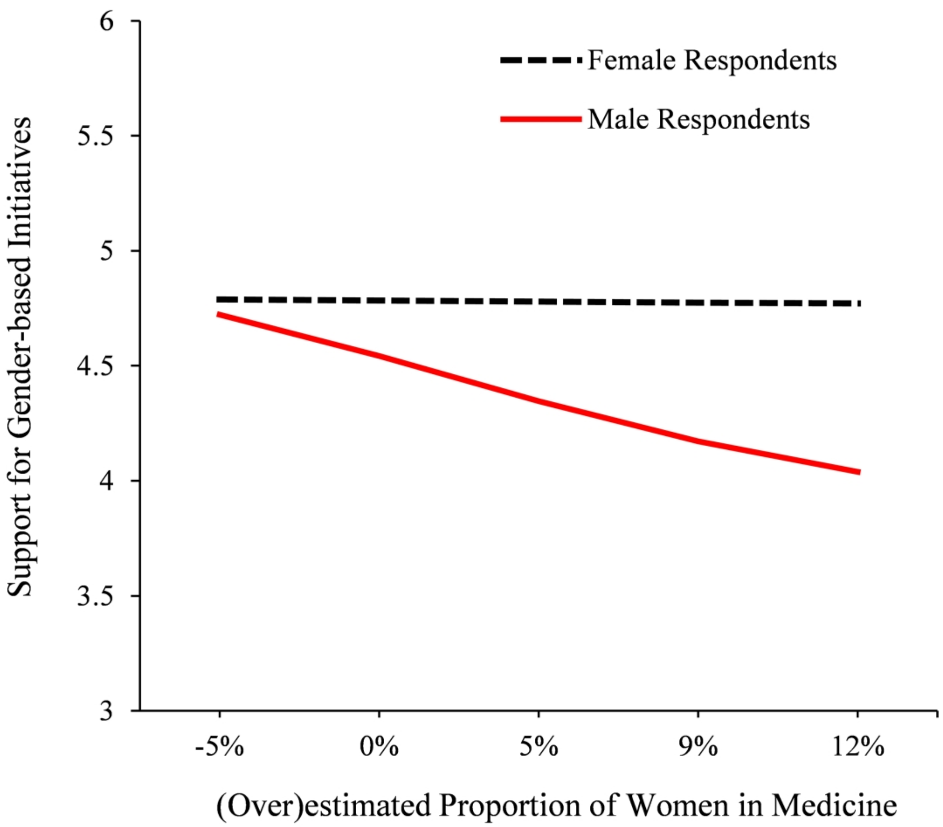

Figure 1. Male and female respondents' (i.e., medical professionals') support for gender-based initiatives in the UK medical profession (1-7 scale), as a function of their estimates of the proportion of women in medicine. Positive values on the x-axis reflect an overestimation of women's representation. Female respondents' estimates were unrelated to their level of support (B = .00, 95% CI = -.02 to .02, p = .92). By comparison, male respondents' tendency to overestimate the proportion of women in medicine predicted significantly less support for gender-based initiatives (B = -.04, 95% CI = -.06 to -.02, p < .001; overestimation*respondent-gender interaction, B = -.04, 95% CI = -.07 to -.01, p = .01, ΔR^2 = .02 for the addition of interaction term, F(1,372) = 6.48, p = .01, f^2 = .02).

118x103mm (600 x 600 DPI)

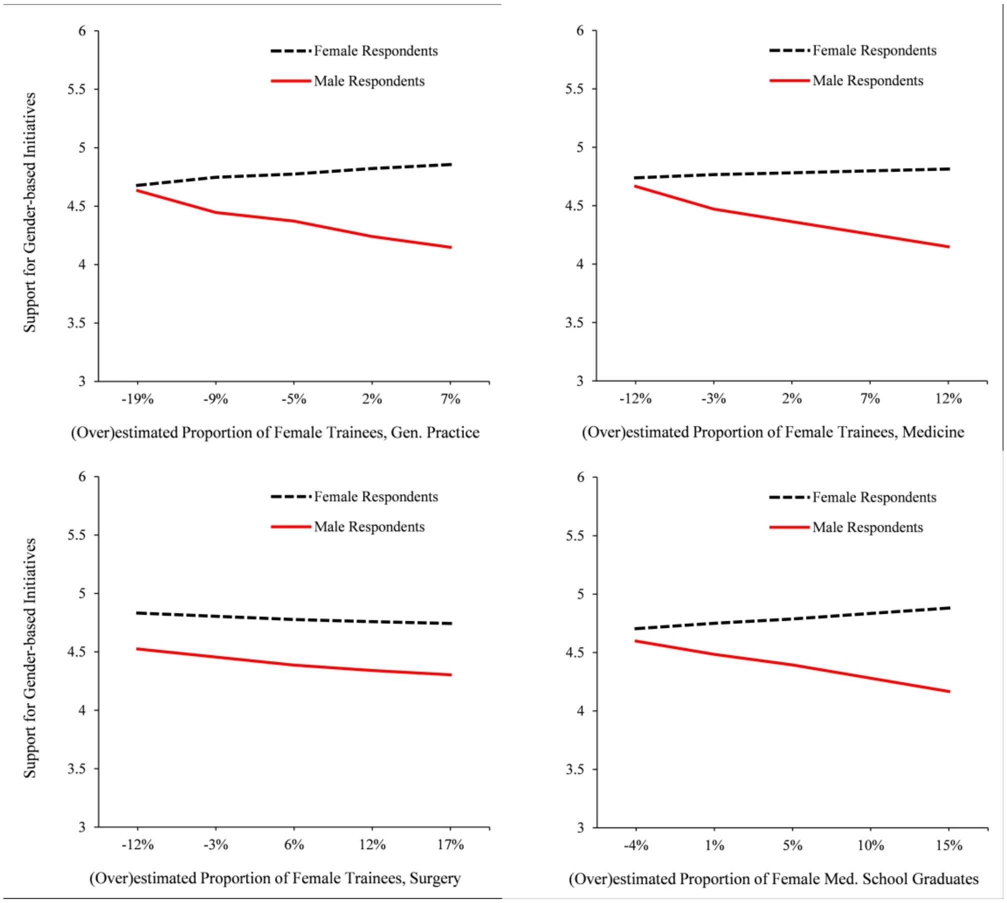

Figure 2. Male and female respondents' (i.e., medical professionals') support for gender-based initiatives in the UK medical profession (1-7 scale), as a function of their estimates of the proportion of: (i) female trainees in general practice, (ii) medicine, and (iii) surgery, and (iv) female medical school graduates. Positive values on the x-axis reflect an overestimation of women's representation in that area/role. In the areas of general practice and medicine, and regarding medical school graduates, female respondents' estimates were unrelated to their level of support, yet male respondents' tendency to overestimate the representation of women in these areas/roles predicted significantly less support for gender-based initiatives. In surgery, neither women's nor men's estimates of female trainees predicted level of support.

211x189mm (600 x 600 DPI)

STROBE 2007 (v4) Statement—Checklist of items that should be included in reports of *cross-sectional studies*

Section/Topic	Item #	Recommendation	Reported on page #
**Title and abstract**	1	(*a*) Indicate the study's design with a commonly used term in the title or the abstract	1
		(*b*) Provide in the abstract an informative and balanced summary of what was done and what was found	3-4
**Introduction**			
Background/rationale	2	Explain the scientific background and rationale for the investigation being reported	5-7
Objectives	3	State specific objectives, including any prespecified hypotheses	6-7
**Methods**			
Study design	4	Present key elements of study design early in the paper	1,3,7
Setting	5	Describe the setting, locations, and relevant dates, including periods of recruitment, exposure, follow-up, and data collection	3,7
Participants	6	(*a*) Give the eligibility criteria, and the sources and methods of selection of participants	7
Variables	7	Clearly define all outcomes, exposures, predictors, potential confounders, and effect modifiers. Give diagnostic criteria, if applicable	3,7-8
Data sources/ measurement	8*	For each variable of interest, give sources of data and details of methods of assessment (measurement). Describe comparability of assessment methods if there is more than one group	7-8
Bias	9	Describe any efforts to address potential sources of bias	6,7,8,15
Study size	10	Explain how the study size was arrived at	7
Quantitative variables	11	Explain how quantitative variables were handled in the analyses. If applicable, describe which groupings were chosen and why	7-17
Statistical methods	12	(*a*) Describe all statistical methods, including those used to control for confounding	9, 10-17
		(*b*) Describe any methods used to examine subgroups and interactions	9,10,12-17
		(*c*) Explain how missing data were addressed	9
		(*d*) If applicable, describe analytical methods taking account of sampling strategy	n/a
		(*e*) Describe any sensitivity analyses	7
**Results**			

For peer review only

Participants	13*	(a) Report numbers of individuals at each stage of study—eg numbers potentially eligible, examined for eligibility, confirmed eligible, included in the study, completing follow-up, and analysed	7
		(b) Give reasons for non-participation at each stage	7
		(c) Consider use of a flow diagram	
Descriptive data	14*	(a) Give characteristics of study participants (eg demographic, clinical, social) and information on exposures and potential confounders	7
		(b) Indicate number of participants with missing data for each variable of interest	7
Outcome data	15*	Report numbers of outcome events or summary measures	7
Main results	16	(*a*) Give unadjusted estimates and, if applicable, confounder-adjusted estimates and their precision (eg, 95% confidence interval). Make clear which confounders were adjusted for and why they were included	9-17
		(*b*) Report category boundaries when continuous variables were categorized	n/a
		(*c*) If relevant, consider translating estimates of relative risk into absolute risk for a meaningful time period	n/a
Other analyses	17	Report other analyses done—eg analyses of subgroups and interactions, and sensitivity analyses	7,16-17
**Discussion**			
Key results	18	Summarise key results with reference to study objectives	17-18
Limitations	19	Discuss limitations of the study, taking into account sources of potential bias or imprecision. Discuss both direction and magnitude of any potential bias	18-19
Interpretation	20	Give a cautious overall interpretation of results considering objectives, limitations, multiplicity of analyses, results from similar studies, and other relevant evidence	17-18
Generalisability	21	Discuss the generalisability (external validity) of the study results	20-21
**Other information**			
Funding	22	Give the source of funding and the role of the funders for the present study and, if applicable, for the original study on which the present article is based	25

*Give information separately for cases and controls in case-control studies and, if applicable, for exposed and unexposed groups in cohort and cross-sectional studies.

Note: An Explanation and Elaboration article discusses each checklist item and gives methodological background and published examples of transparent reporting. The STROBE checklist is best used in conjunction with this article (freely available on the Web sites of PLoS Medicine at http://www.plosmedicine.org/, Annals of Internal Medicine at http://www.annals.org/, and Epidemiology at http://www.epidem.com/). Information on the STROBE Initiative is available at www.strobe-statement.org.

For peer review only
